# Age-congruency and contact effects in body expression recognition from point-light displays (PLD)

**DOI:** 10.7717/peerj.2796

**Published:** 2016-12-13

**Authors:** Petra M.J. Pollux, Frouke Hermens, Alexander P. Willmott

**Affiliations:** 1 School of Psychology, University of Lincoln, Lincoln, United Kingdom; 2 School of Sport and Exercise Science, University of Lincoln, Lincoln, United Kingdom

**Keywords:** Body expression recognition, Age congruency, Contact effect

## Abstract

Recognition of older people’s body expressions is a crucial social skill. We here investigate how age, not just of the observer, but also of the observed individual, affects this skill. Age may influence the ability to recognize other people’s body expressions by changes in one’s own ability to perform certain action over the life-span (i.e., an own-age bias may occur, with best recognition for one’s own age). Whole body point light displays of children, young adults and older adults (>70 years) expressing six different emotions were presented to observers of the same three age-groups. Across two variations of the paradigm, no evidence for the predicted own-age bias (a cross-over interaction between one’s own age and the observed person’s age) was found. Instead, experience effects were found with children better recognizing older actors’ expressions of ‘active emotions,’ such as anger and happiness with greater exposure in daily life. Together, the findings suggest that age-related changes in one own’s mobility only influences body expression categorization in young children who interact frequently with older adults.

## Introduction

Recognition of the emotional state and intentions of others is a crucial skill for social interaction. Cues from facial expressions, prosody and body movements all provide valuable information for rapid interpretation of the social environment and allow for the adjustment of one’s own behaviour to enhance social survival. Whereas most insights about the processing of social cues have been obtained from facial expressions, investigations of body posture and movement have become more prevalent. Cues from body movements and posture convey important information about the emotional state of people seen from a distance (De Gelder, 2009) and about the intensity of the emotion (Wallbott, 1998). Moreover, information from body posture and movements generally improve emotion recognition compared to when only the face is visible. Similarly, bodily cues that are inconsistent with the facial expression alter or slow down affective judgements (Ambady & Rosenthal, 2008; Van der Stock, Righart & de Gelder, 2007; Avieser et al., 2008). Evidence for the role of body cues for emotion recognition has been found for posture (Coulson, 2004), gestures (De Meijer, 1989) arm/hand/head movements (Dael, Mortillaro & Scherer, 2012) and kinematic characteristics of movements, such as velocity, amplitude, fluidity of movements, or jerk movements (Bernhardt & Robinson, 2007; Castellano, Villalba & Camurri, 2007; Glowinski et al., 2011). For example, Coulson (2004) found similar emotion recognition in avatars from static emotional postures and static emotional faces for some basic emotions. This emotion specificity in postures has also been found for movements in dynamic stimuli. For instance, Dael, Mortillaro & Scherer (2012) showed that specific movements and orientations of the hands, arms, trunk and head of actors clustered uniquely for some emotions, such as hot anger, amusement and pleasure. Moreover, emotional state can be predicted above chance from motion features such as amplitude, speed and fluidity alone when actors make identical arm movements in different (acted) emotional states (Castellano, Villalba & Camurri, 2007).

The above features of emotion recognition from body cues all discount any effects of the age of the observer, and of the actor. For face processing, however, a specific interaction has been found between one’s own age and the age of the observed person, an effect known as the own-age bias (Rhodes & Anastasi, 2012). In this bias, faces of other people who are of a similar age as the observer are processed faster and more accurately. Participants tend to remember own-age faces better (He, Ebner & Johnson, 2011), are more distracted by own-age faces when engaged in an ongoing task (Ebner & Johnson, 2010), and look at own-age faces for longer (Ebner, He & Johnson, 2011). Moreover, own-age faces have been found to enhance activity in the prefrontal cortex compared to other-age faces. This enhanced activity has been related to higher interest and stronger engagement of self-referential processing when viewing own-age faces (Ebner et al., 2013).

The own-age effect in face identity judgments has been explained by enhanced perceptual expertise from more extensive experience with people of the same age category (Harrison & Hole, 2009; Rhodes & Anastasi, 2012; Wiese, Komes & Schweinberger, 2012; Wiese et al., 2013). For example, in contrast to a control group with only infrequent contact with older adults, young geriatric nurses showed no own-age bias towards faces of older adults (Wiese et al., 2013). Similarly, young teachers with frequent contact with children did not show the own-age memory bias observed in a standard control group (Harrison & Hole, 2009).

While face identity detection tasks consistently demonstrate an own-age bias, the bias is less often observed for expression recognition. Some studies found age-congruence effects in expression categorization (Malatesta et al., 1987) and emotional state recognition (pleasant, unpleasant or no feeling; Riediger et al., 2011), whereas other studies showed no age-congruence effect, neither for facial expression categorization (e.g., Ebner, He & Johnson, 2011; Ebner, Johnson & Fischer, 2012; Ebner et al., 2013) nor for expression intensity judgments (Hühnel et al., 2014). This may suggest that age-related features in faces are more relevant for identity than for expression judgments.

The extent to which the findings about the own-age bias in facial expression recognition can be generalized to body expression recognition is unknown. Body and facial cues for emotion recognition have been suggested to differ functionally and differential underlying neural processes have been proposed (De Gelder et al., 2010). It has been suggested, for example, that compared to facial expressions, body posture and movement convey more important information about action intentions of others and prepare the perceiver for adaptive reactions (De Gelder et al., 2004). Such a proposal is consistent with converging evidence from fMRI and MEG revealing category-specific activation for bodies and faces (Meeren et al., 2008; Atkinson, Vuong & Smithson, 2012). Moreover, findings indicate that only emotional bodies, but not neutral bodies or emotional faces, activate cortical and subcortical motor related structures such as IFG, caudate and putamen (De Gelder et al., 2004; Van de Riet, Grèzes & de Gelder, 2009), consistent with the idea that body expressions activate action-related neural structures.

Own-age biases in body expression recognition are consistent with simulation theories for action understanding and emotion recognition, involving neural structures such as the ‘mirror-neuron system.’ For example, similar responses to actions that were either observed or self-executed have been found for specific brain areas (Rizzolatti & Craighero, 2004; Caligiore et al., 2013; Grafton & Tipper, 2013). An important implication of such findings in the context of the present research is that action understanding can be expected to depend on the observer’s ability to produce the same action (Gallese & Goldman, 1998; Gallese, 2009). Consistent with this idea, motor experience has been shown to be related to imitation and action understanding in early infancy (e.g., Van Elk et al., 2008; Paulus et al., 2011). For example, mu- and beta desynchronizations (an EEG correlate of motor resonance) in 14–16 month old infants were stronger when viewing videos of other babies crawling, an action within the infants’ motor repertoire, or walking, which the infants had no experience with yet (Van Elk et al., 2008). Furthermore, own-age biases were found in the priming of object grasps by hand shapes (Liuzza, Setti & Borghi, 2012). Times to initiate grasps were shorter when own age grasping hands were shown before one’s own grasp, an effect attributed to motor resonance (Liuzza, Setti & Borghi, 2012).

Similar to action observation, observing emotions in others can be expected to give rise to experiencing the same emotion due to overlap in activation of brain representations (Barsalou, Solomon & Wu, 1999; Damasio, 1999; Niedenthal et al., 2005; for comprehensive review, see Heberlein & Atkinson, 2009; Oosterwijk & Barrett, 2014; Mitchell & Phillips, 2015; Winkielman et al., 2015). The role of simulation in body expression recognition has been demonstrated by the enhancement of subjective ratings of one’s own emotional state by either observing, imagining or actually executing the movements associated with specific emotions (Shafir et al., 2013). Moreover, people who suffer from alexythemia (characterized by a reduced ability to perceive one’s own emotions) were found to be less confident in their judgments about the emotional valence of a social scene, suggesting a link between one’s own experience of emotion and the perception of emotional state in others (Lorey et al., 2012). In most models of emotion processing, expression identification involves extensive neural networks where simulation is integrated with perceptual and conceptual processes (Niedenthal et al., 2005; Caligiore et al., 2013; Oosterwijk & Barrett, 2014; Mitchell & Phillips, 2015).

The present study examines whether the own-age bias found for face perception and in action perceptions extends to emotion recognition from body cues. Such a bias may be expected from shared neural representations for action perception and production (Rizzolatti & Craighero, 2004; Caligiore et al., 2013; Grafton & Tipper, 2013) and from changes in postural and motor control across the life-span (e.g., Seidler et al., 2010; Pojednic et al., 2012). As a consequence, emotion recognition from body cues may be enhanced when the age of the observer and the observed person overlaps. The present work specifically focuses on motion cues, as kinematics can be expected to be strongest influenced by age. To isolate motion cues from any other body cues (e.g., body size, gender, race), Point Light Displays (PLDs) were used. These are videos of markers attached to joints in moving people, typically shown as moving white dots on a dark background. PLDs of children, young adults and older adults enacting whole body expressions of six basic emotions were created and were presented to participants of the same three age-groups. Past research has suggested that people can categorize emotions from PLDs with high accuracy, particularly when the actors have been encouraged to exaggerate their whole body responses (Atkinson et al., 2004; Atkinson, Tunstall & Dittrich, 2007).

Two experiments were undertaken. While the observers in Experiment 1 were unaware of the actors’ ages, observers in Experiment 2 were informed about the actors’ ages. This difference tested whether knowing someone’s age group helps in recognizing emotion from PLDs. In both experiments, observers of different age groups were asked to recognize emotions from PLDs of different age groups. Experiment 1 compared children, young adults and older adults, while Experiment 2 compared children and younger adults. The role of experience with certain age groups (Harrison & Hole, 2009; Rhodes & Anastasi, 2012; Wiese, Komes & Schweinberger, 2012; Wiese et al., 2013) was examined by incorporating a measure of contact with other age groups in the analysis.

If body expression categorization is characterized by own-age biases, the results should confirm an age-congruency effects, characterized by higher accuracy for PLDs of actors who are of the same age-group as the viewer. Potential interactions with previously reported influences on body expression categorization, such as the emotion expressed (e.g., Atkinson et al., 2004) will be explored in a second analysis.

## Experiment 1

Experiment 1 investigated whether an own-age bias is found for body expression recognition from PLDs when no information is given about the actors’ ages. In the presence of such a bias, recognition performance should show a cross-over interaction between the observer’s age and the observed person’s age (Malatesta et al., 1987), with strongest performance when the age of the observer and the actor overlap and weaker performance otherwise.

### Methods

#### Participants

Participants from the different age groups were recruited by different means. Fifty-six young adults (23 males, age = 19.4 ± 0.99 years; 33 females, age = 19.6 ± 2.8 years) were recruited via the Subject Pool of the School of Psychology at the University of Lincoln. Thirty-four older adults aged 65 or older (15 men, age = 73.6 ± 5.6 years, 19 women, age = 73.7 ± 4.8 years) were recruited locally via advertisements. The cognitive status of older participants was assessed with the Mini Mental State examination. All older participants obtained a score of 28 or more out of a maximum score of 30, thereby exceeding the criterion score that would indicate cognitive decline (24). Children (40 boys and 34 girls) aged between 6 and 10 years old (7.6 ± 0.13), were recruited and tested during a ‘Summer Science’ week organised by the School of Psychology. The sample sizes for each age-category of participants was determined by (1) the number of participants who signed up for the study via the Subject Pool (young adults), (2) the number of older adults who responded to the local advertisements, and (3) the number of children who participated in the Summer Science week. The sample was collected as a whole without any intermediate statistical analysis and therefore optional stopping was avoided.

Informed written consent was obtained from participants and from the parents of the children. All participants had normal or corrected to normal vision. Ethical approval was obtained from the School of Psychology Research Ethics Committee, University of Lincoln (Reference number: 131218-pp). All procedures complied with the British Psychological Society “Code of Ethics and Conduct” and with the World Medical Association Helsinki Declaration as revised in October 2008.

#### Materials

Seventy-two clips of amateur actors enacting whole body expressions of six expressions (happy, sad, anger, fear, surprise and disgust) and three actions (kicking, throwing and digging) were recorded in the Biomechanics Laboratory in the School of Sport and Exercise Science at the University of Lincoln (http://www.lincoln.ac.uk/home/sport/). Local professional and amateur actors (e.g., students from the drama school of the University of Lincoln or members of local amateur theatre groups) performed the recorded movements. Actors consisted of 4 children (two boys, 8 and 9 years old and two girls, 9 and 10 years old), 4 older adults (2 women, 70 and 72 years of age and 2 men, 72 and 74 years of age) and 4 young adults (2 women, 21 years old and 2 men, 21 and 22 years old). Informed consent was obtained from all actors and from the parents of the children.

Acting of whole body expressions was directed by a professional theatre and film director/actor (Ben Keaton) to optimise performance of the amateur actors. Each actor was instructed to start and finish in a neutral body position, with legs slightly apart and arms resting to the side of the body. The scenario approach was used during recording of the body expressions (Wallbott, 1998; Atkinson et al., 2004; Ross, Polson & Grosbras, 2012; Gunes et al., 2015) to ensure that the PLDs displayed clearly discernible and high intensity expressions (Atkinson et al., 2004). Each scenario, describing a situation that is likely to induce a strong emotional response, was outlined by the director at the beginning of each recording. Actors were encouraged to imagine how they would feel in the situation described and to act out how they would express this feeling with their whole body.

Scenarios varied slightly for the three age groups to ensure relevance of the theme. For example, one adult scenario for ‘hot anger’ describes a situation where one of the drivers of two cars, involved in a minor car crash, falsely accuses the other driver (the actor) of irresponsible and bad driving, despite the fact that the actor was innocent. For children, one scenario describes a situation where the child actor is falsely accused by the parent of causing a fight with a sibling or close friend.

Motion capture was undertaken using ten Raptor cameras sampling at 150 Hz through Motion Analysis Cortex (Motion Analysis Corporation, Santa Rosa, CA, USA). The three-dimensional trajectories of retroreflective markers attached to 42 anatomical landmarks on the head, trunk, arms and legs were recorded. These data were used as input for custom code in MATLAB (Mathworks, Natick, MA, USA) that generated the final point-light displays (PLDs) of white dots moving against a black background. Fifteen points were shown: the centre of the head, the base of the neck (at the level of the suprasternale), the base of the spine (at approximately the level of the fifth lumbar vertebra), and the left and right shoulder, elbow, wrist, hip, knee and ankle joint centers. The motions were smoothed using a zero-lag, fourth-order, low-pass Butterworth filter with a cut-off frequency of 6 Hz. The final clips were five seconds in duration and displayed whole body motions from the frontal viewpoint.

#### Procedure

The experiment was conducted with a laptop (HP Pavilion TouchSmart15). Before the experimental trials, there was a practice block of nine trials. This practice block consisted of clips of three different types of actions (kicking, throwing and digging) and six additional practice clips for each expression. At this stage, the adult participants were also introduced to the confidence rating requested after each clip. For young and older adults, the action and practice trials were followed by the main task consisting of 72 trials. Participants provided their responses verbally, after which they were immediately entered into a computer by the experimenter. The task was therefore paced by the speed of vocal responses given by the participants. Each trial started with the word “Ready?” presented in the centre of the screen. Participants were required to indicate verbally that they were ready to continue. The clip was presented for 4 s, followed by a screen displaying the six response options. After the verbal response of the participant (entered by the experimenter), adults were asked to give a confidence rating of their answer: “How confident are you? 1 = not confident at all, 9 = extremely confident”. Once the rating was entered, the “Ready?” screen for the next trial appeared. After completion of the experiment, participants filled in a brief questionnaire including questions about the average number of hours per month spent with children, young adults or older adults. Although confidence ratings for emotion recognition in the PLDs were collected, these ratings were not directly relevant for the aim of the present study and are therefore not further described.

While for young and older adults, a large number of stimuli could be presented, testing time was restricted for children (with a maximum testing time of around 15 min per participant, imposed by the ethics approval). The number of trials was therefore reduced for children to a total of 18 trials, which also allowed for some time to introduce the emotion labels to the children. In this introduction, children were presented with six slides containing cartoon figures with high intensity facial expressions and were asked to label the expression with the following question: “What are they feeling?” The intended expressional label or a synonym was mentioned by all children for five out of six expressions (e.g., ‘cross’ for angry or ‘scared’ for fearful). The exception was disgust, where some children used an alternative way to describe the expression, e.g., “doesn’t like it” or “yuck!” In these instances, the experimenter would introduce the label ‘disgust’ to the child but their own synonyms were also accepted during the testing sessions.

Children were presented with 18 clips, which were of one female child, one young adult female and one older adult female. Selection was based on categorization responses of the young adult participants who were tested before data collection of the children. To optimize the youngest children’s performance, models with the highest accuracy score were selected. Although accuracy to PLDs for male and female actors were not significant different (t(55) = 1.25; *p* = 0.216), the highest mean accuracy scores were mostly associated with female models. After the children completed the experiment, the parent filled in the questionnaire including questions about how much time the child spends with people of own and other age-groups per month.

### Results

#### Body expression recognition

Statistical analyses applying generalized linear mixed models (GLMM) were conducted with the lme4 package in R. Generalized mixed models, which account for random factors while modelling variance associated with experimental factors, have been suggested to be more appropriate for analysis of categorical responses compared to standard ANOVA models (Jaeger, 2008). For the analysis of accuracy, responses were coded as correct or incorrect. Two predictive models were tested against each other. Model 1 focused on the key prediction for this study: If own-age biases influence body expression categorization accuracy, then a significant interaction between the age category of the viewer (‘Age-Viewer’; children, young adults, older adults) and the age category of the actor (‘Age-Actor’; children, younger adults, older adults) could be expected. This interaction effect could be expected to be characterized by more correct responses for body expressions posed by actors who belong to the same age category as the viewer. The complexity of the model was increased in Model, 2 where factors that have previously been shown to influence emotion recognition were added: Type of Emotion (anger, happy, fear, sad, disgust and surprise; Atkinson et al., 2004; Atkinson, Tunstall & Dittrich, 2007) and Gender of the viewer (Kret & De Gelder, 2012). In both Models, variability between participants (‘Subjects’) and the different video clips (‘Item’) were included as random factors.

**Figure 1:**
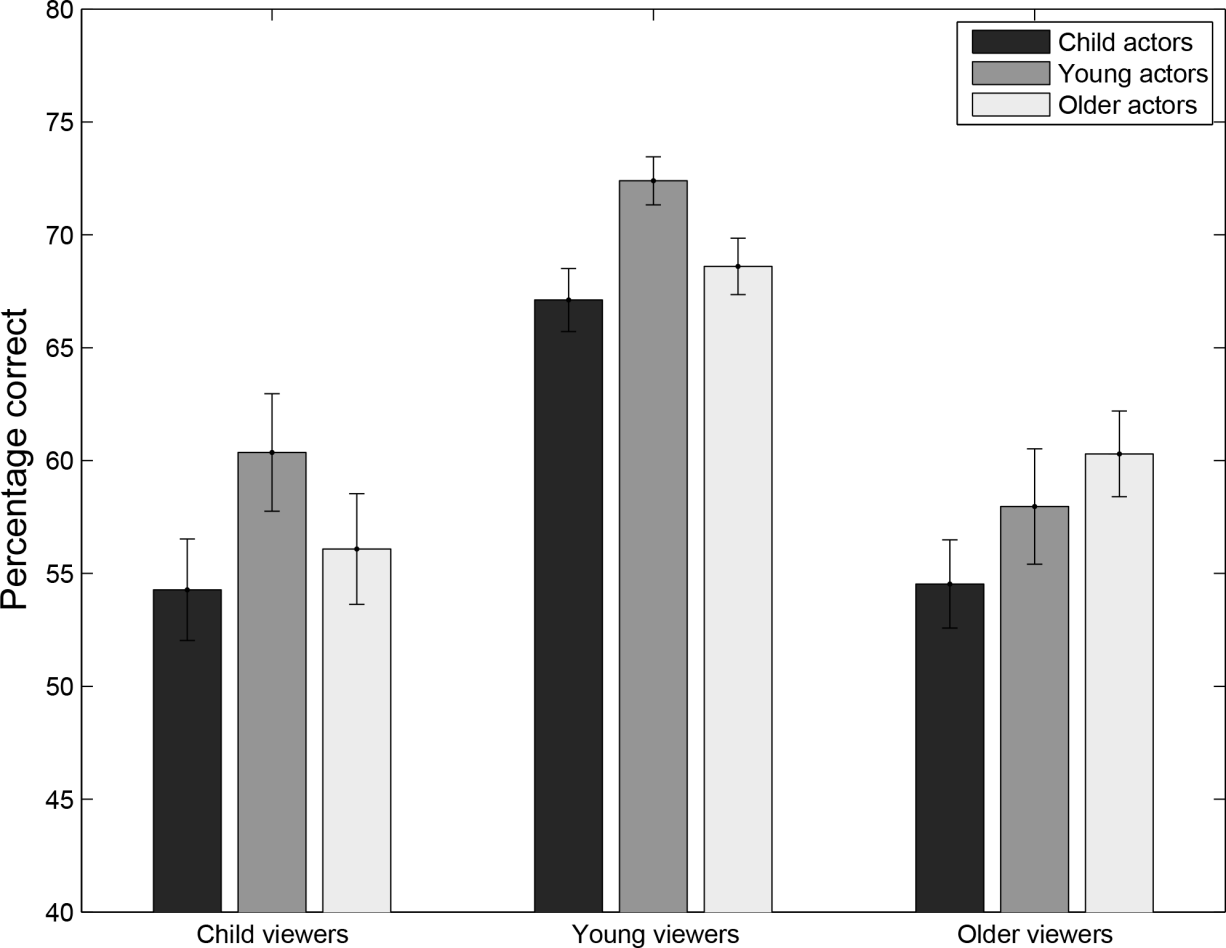
Experiment 1: percentage correct responses for viewers (Young viewers, Older viewers, Child Viewers) as a function of Age-of-Actor: (Young actors, Older actors, Children).

**Table 1:** Experiment 1: results of a mixed models analysis. Generalized linear mixed model fit by maximum likelihood (Laplace Approximation) [‘glmerMod’], Family: binomial (logit): Formula Model 1: proportion correct responses ∼agegroup + ageactor + agegroup * ageactor + (1 | su) + (1 | itemnr), Model 2: proportion correct responses ∼agegroup + ageactor + agegroup * ageactor + emotion + emotion * agegroup + (1 | Subjects) + (1 | Items).

	Subjects and items	
	Estimate (SE)	*p*
**Model 1**		
***Fixed factors:***		
Intercept	1.93 (0.42)	<0.001
Age-Viewer	−.64 (.12)	***<.001***
Age-Actor	−.24 (.19)	.19
Age-Actor × Age-Viewer	.11 (.05)	***.036***
*AIC*	*8,664*	
*BIC*	*8,706*	
*Random factors*	*Variance (SD)*	
Subjects (Intercept)	0.17 (0.41)	
Items	1.32 (1.14)	
**Model 2**		
***Fixed effects:***		
Intercept	3.73 (.46)	<.001
Age-Viewer	−.81 (.15)	***<.001***
Age-Actor	−.25 (.15)	.11
Emotion	−.49 (.073)	***<.001***
Gender	−.03 (.08)	.72
Age-Actor × Age-Viewer	.11 (.005)	***.032***
Emotion × Age Viewer	.04 (.023)	*.056*
*AIC*	*8,632*	
*BIC*	*8,695*	
*Random factors*	*Variance (SD)*	
Subjects (Intercept)	0.16 (0.4)	
Items	0.77 (0.88)	

Figure 1 provides an overview of the results, by plotting the average accuracy across participants after first averaging across trials. Figure 1 suggests that while performance was slightly better for their own age group in young and older adults, children did not shown an own age advantage. These results, however, do not include effects of the acted emotion and gender of the viewer. They also do not take into account the variability across items. In order to better evaluate the relative contribution of these effects, general linear models were fitted to the data and compared (Table 1).

The random factors in these models capture the variability in the data across participants and items (random intercepts). The Akaike information criterion (AIC) was used to compare the relative contribution of these two random factors to the two Models (Vrieze, 2012): Table 2 shows that AIC values are reduced when a random factor for ‘Items’ is included compared to when only a random factor for subjects is included, suggesting that the variation across items influences body expression accuracy substantially. AIC values were slightly lower when both Subjects and Items were included as random factor (AIC = 8,632) compared to when only Items were included (AIC = 9,379). Both random factors were therefore included in all Mixed Linear Model analyses reported here. AIC and BIC values were smaller for Model 2, suggesting a better fit when the Model includes the fixed factors Emotion and Gender.

**Table 2:** Experiment 2: results of mixed models analysis. Generalized linear mixed model fit by maximum likelihood (Laplace Approximation) [‘glmerMod’], Family: binomial (logit): Formula Model 1: proportion correct responses ∼agegroup + ageactor + agegroup * ageactor + (1 | su) + (1 | itemnr), Model 2: proportion correct responses ∼agegroup + ageactor + agegroup * ageactor + emotion + emotion * agegroup + (1 | Subjects) + (1 | Items).

	Subjects and items	
	Estimate (SE)	*p*
**Model 1**		
***Fixed factors:***		
Intercept	2.45 (.61)	<.001
Age-Viewer	−1.1 (.34)	*** <.001***
Age-Actor	−.33 (.24)	.19
Age-Actor × Age-Viewer	.23 (.14)	.10
*AIC*	*4,606*	
*BIC*	*4,644*	
*Random factors*	*Variance (SD)*	
Subjects (Intercept)	0.49 (0.71)	
Items	1.55 (1.24)	
**Model 2**		
***Fixed effects:***		
Intercept	4.4 (.72)	<.001
Age-Viewer	−1.5 (.43)	***<.001***
Age-Actor	−.32 (.22)	.14
Emotion	−.57 (.10)	***<.001***
Gender	−.03 (.15)	.81
Age-Actor × Age-Viewer	.23 (.14)	.10
Emotion × Age-Viewer	.09 (.06)	.14
*AIC*	*4,577*	
*BIC*	*4,634*	
*Random factors*	*Variance (SD)*	
Subjects (Intercept)	0.48 (0.69)	
Items	0.9 (0.95)	

##### Model 1 (confirmatory analysis): own age bias

A significant interaction effect between Age-Viewer × Age-Actor was found for Model 1. While such an interaction may suggest an own age bias in the data, a pattern of results consistent with the own age bias was only found for younger and older adults, who both showed a trend towards superior performance for their own age group (Fig. 1). Children, however, showed strongest performance for young adult actors, suggesting that although a significant interaction was found, the data do not provide strong evidence for an own age bias. To test the statistical significance of these observed patterns, post-hoc tests were conducted for each of the three age groups separately, testing the effect of the age of the actor. Importantly, no effect of actor’ age-group was found, neither for young adult viewers (*z* =  − 0.87, *p* = 0.38), nor for older adult viewers (*z* =  − 0.42, *p* = 0.68) or child viewers (*z* =  − 0.38, *p* = 0.71). To further explore possible explanations for the interaction, the effect of viewer’s age and actor’s age was analysed separately for pairs of viewer age-group. The results show that the strongest interaction was found when only young adults and children were included in the analysis (*z* = 1.81, *p* = 0.071), a weaker interaction when only older adults and children were included (*z* = 1.34, *p* = 0.17), and an even weaker interaction when older adults and younger adults were considered (*z* = 0.85, *p* = 0.39). Importantly, none of these interactions effects reached statistical significance. The overall interaction effect therefore depends on the comparison of all three groups of viewers and all three age groups of age actors. The strongest interaction in direct group comparisons was found between young adults and children, but looking at the data in Fig. 1, this interaction is not linked to the predicted cross-over interaction. It is therefore more likely that the interaction effect is driven by differences in performance between viewer groups. Significant effects of viewer age-group (including all three viewer age-groups) were indeed found for PLDs of young adult actors (*z* =  − 7.8, *p* < 0.001), older adult actors (*z* = 3.13, *p* = 0.0018) and child actors (*z* = 5.67, *p* < 0.001). Post-hoc comparisons of viewer age-groups, separately for each actor-age group showed that while younger adult viewers outperformed both older adult viewers and children for all three actor age-group conditions (*p* ≤ 0.001), older adult viewers performed better compared to child viewers for PLDs of young adult actors only (*p* = 0.038), whereas this difference was not significant for PLDs of older adult actors and child actors (*p* ≥ 0.23).

So far we have only considered random intercepts. However, Barr et al. (2013) argue that including random slopes could be beneficial for generalizability of the Model. For our confirmatory analysis, we therefore determined whether inclusion of random slopes would significantly improve the fit of Model 1. Chi-square test results showed however, that the additional degrees of freedom introduced by the random slopes did not significantly improved the Model fit (Chi square (*df* = 2) = 1.34; *p* = 0.51).

##### Model 2 (exploratory analysis)

Model 1 only takes into account the age of the actor and the age of the observer. Stimuli, however, also varied in the emotion they conveyed, and we also recorded the gender of the viewer. The effects of these factors were examined in Model 2. This model revealed statistically significant contributions of Age-Viewer, Emotion and Emotion × Age-Viewer, whereas the effect of Gender-Viewer was not significant. The Age-Viewer × Age-Actor interaction, that was significant in Model 1, remained and its associated statistics were largely unaffected by the inclusion of emotion and Gender-Viewer.

**Figure 2:**
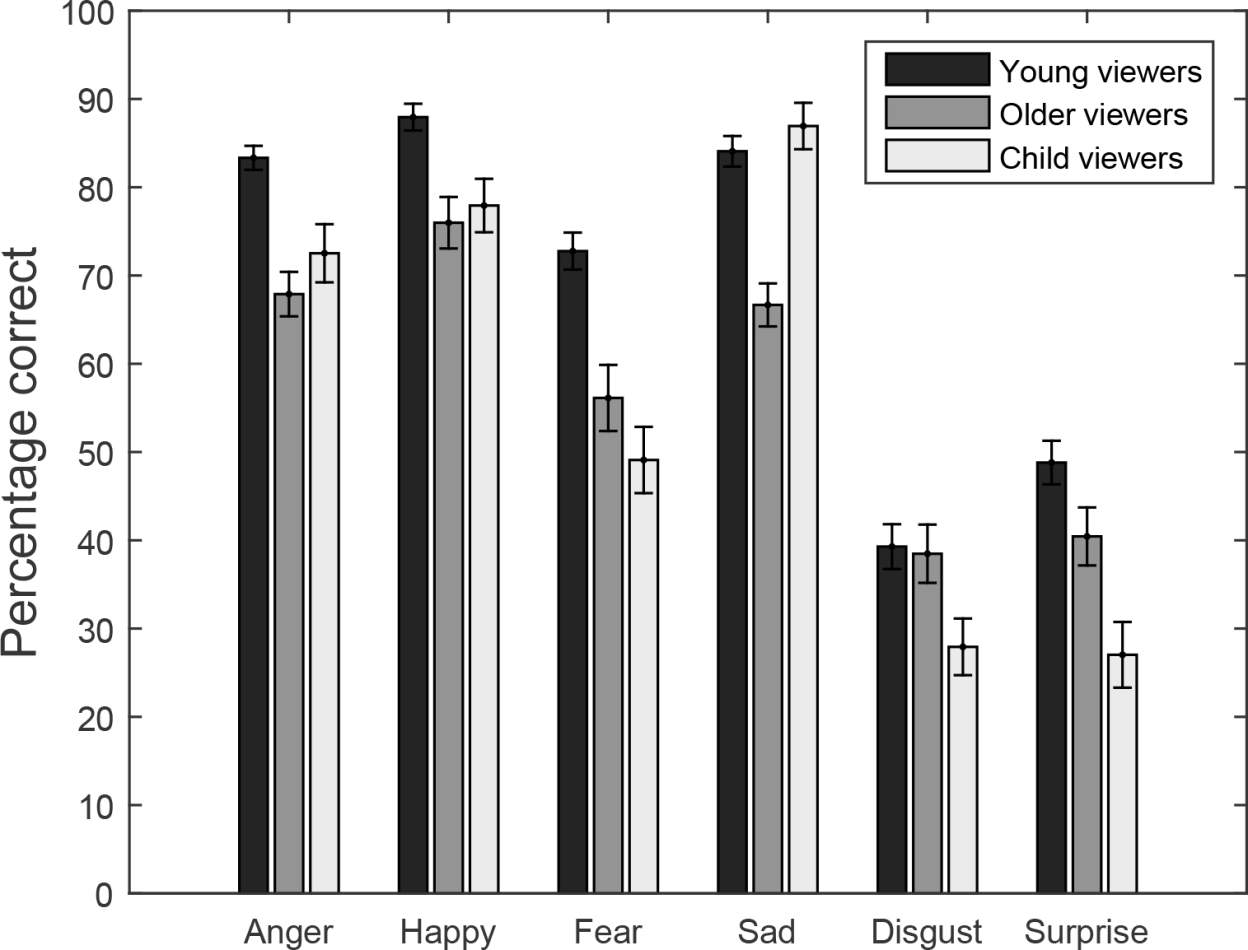
Percentage correct responses for viewers (Young viewers, Older viewers, Child Viewers) as a function of body expression (Anger, Happy, Fear, Sad, Disgust and Surprise).

Figure 2 explores the nature of the effects of emotion and the interaction with the age of the viewer. These data suggest that anger, happiness, fear and sadness were more easily recognized than disgust and surprise. Children were good a recognizing sad emotions (better than the older participants), while they were performing worse for disgust and surprise. Bonferroni corrected (15 comparisons, critical *p*-value = 0.00333) post-hoc analyses on subsets of the data (e.g., only happy and sad stimuli) using the same Model 2 for the analysis, showed significant differences between Anger and Disgust, Anger and Surprise, Happy and Disgust, Happy and Surprise, Fear and Disgust, Fear and Surprise, Sad and Disgust, and Sad and Surprised (all *p*’s <0.001). The main effect of emotion for the Fear-Disgust comparison was accompanied by a significant interaction with age of the viewer (*p* = 0.0029), reflecting the particular high accuracy in children in the Sad condition and low accuracy for Disgust stimuli. Comparison of younger and older adults showed a significant effect of emotion (*p* < 0.001) and an interaction between emotion and age-group (*p* < 0.001). Bonferroni corrected comparisons between younger and older adults for each of the emotions showed that older viewers were significantly worse for Fear and Sad stimuli (*p* < 0.001). A comparison between young adults and children showed an effect of emotion, age-group, but no emotion by age-group interaction, reflecting the overall worse performance for children, and differences in the emotions overall. A comparison between older adults and children showed a significant emotion by age-group interaction, but no main effect of emotion. Interestingly, this interaction appears to be driven by differences for the Fear emotion alone (*p* = 0.0017), when gender of the viewer, and the interaction between age of the viewer and age of the actor are taken into account.

##### Contact

Potential associations between contact and accuracy in body expression categorization were first explored with correlations between the estimated number of hours per month (HPM) spent with children, younger adults and older adults and the average percentage correct responses (collapsed over expression) to body expressions posed by children, young adults, and older adults. These analyses revealed only one borderline significant positive linear relationship: for child viewers only, the amount of contact with older adults correlated positively with accuracy of children for clips of older actors (Pearson’s *r* = 0.23, *p* = 0.05). All remaining correlations in all three age groups were not significant. Moderated Linear Regression (Hayes, 2013) was subsequently used to explore if the age-group of the viewer is a significant moderating factor for the relationship between contact with older adults and percentage correct categorizations of body expressions posed by older adults. Age-group of the viewer was coded as a binary variable, contrasting child viewers with adult viewers. Age-group Viewer and children’s contact with older adults (estimated average hours of contact per month (HPM-older adults)) were entered as predictors for percentage correct responses to expression posed by older adults in the first step of the hierarchical model (Model 1). After centring the means to reduce collinearity, the interaction term (Age-group Viewer × HPM-older adults) was added in the second step (Model 2). Model 1 accounted for a significant amount of variance in children’s ability to recognize body expression posed by older actors (*R*^2^ = 0.09, F(2,161) = 7.9, *p* = 0.001). Addition of the interaction effect in Model 2 revealed a significant change in *R*^2^ (*R*^2^ change = 0.025, F(1, 160) = 4.4, *p* = 0.036), suggesting a moderating factor of Age-group Viewer on the relationship between children’s contact with older adults and their percentage correct responses to body expression posed by older adults. Additional analysis of the slopes separately for the age groups showed a significant positive slope for children (*B* = 0.41; *t* = 1.98, *p* = 0.05) which was not significant for adults (*B* =  − 0.057; *t* = 0.1.3, *p* = 0.13).

### Discussion

Experiment 1 set out to investigate whether emotion recognition in other people’s body movements (shown as point light walker, PLDs) shows an own-age bias, where highest performance can be expected when the age of the observer and the age of the observed person agree. An own age bias was hypothesized from the shared neural representation of action performance and observation, and the age-related constraints on one’s own body movements. The findings of Experiment 1 revealed a significant interaction effect between age of the viewer and of the actor, yet this interaction did not reflect the pattern of results predicted by an own-age biases (i.e., higher accuracy for one’s own age group). Children, in particular, showed better performance for young adult actors than for child actors, although none of these within group comparisons reached statistical significance. The finding that body expression recognition was not clearly characterized by age-congruency in the present study effects seems to be in line with the facial expression literature, where the majority of studies found no evidence for an own-age bias in facial expression processing (Ebner, He & Johnson, 2011; Ebner, Johnson & Fischer, 2012; Ebner et al., 2013; Hühnel et al., 2014, but see Malatesta et al., 1987; Riediger et al., 2011). Analysis of Model 2 further revealed that in line with previous findings, young adults outperformed both older adults (e.g., Insch et al., 2015) and children (Ross, Polson & Grosbras, 2012) and performance depended on the emotion considered. These findings will be discussed in more detail in the General Discussion.

Part of our findings could be explained from exposure to the different age groups. We found a relationship between children’s contact with older people and their ability to recognize expressions in older actors, suggesting that regular contact with older adults may facilitate children’s ability to recognize emotional state of older people. Associations between contact and recognition performance have been consistently reported for face identify recognition (Harrison & Hole, 2009; Wiese, Komes & Schweinberger, 2012; Wiese et al., 2013), whereas contact associations with emotion expression recognition are less commonly found. In the one study that found an association between amount of contact and emotion recognition, young adults demonstrated lower accuracy in facial expression categorization of older adults if they had more contact with their own age group, but the reverse effect was not observed in older adults (Ebner & Johnson, 2009). They argued that high interest of young adults in their own-age group may be associated with less interest in older adults, resulting in a reduced ability to recognize older people’s emotional state. In contrast to these findings, the current results suggest that body expression recognition in young adults is not influenced by the amount of contact they have with people of their own age or with other age-groups.

One factor which complicates direct comparisons between evidence from the facial expression literature and the results of the current study is that recognition of the ages of the actors is less obvious for PLDs than for faces (Fölster, Hess & Werheid, 2014). Knowledge of the actor’s age is likely to be beneficial for identification of certain body expressions. For instance, knowing that the observed actor may have reduced movement velocity due to old age could be beneficial when deciding on more ambiguous expressions of ‘active’ emotions, such as anger (Dael, Mortillaro & Scherer, 2012). In addition, own-age biases have been linked to participants’ awareness of the actors’ ages (He, Ebner & Johnson, 2011). To investigate whether being aware of the age of the actor in the stimulus influences performance, Experiment 2 replicated the paradigm of Experiment 1 in young adults and children (the two age groups from which recruitment was most straightforward), but now participants were informed about the actor’s age before presentation of the stimulus.

## Experiment 2

Experiment 2 aims to investigate whether knowing the age of the actors in PLDs influences how body expressions of emotions are perceived by young adults and children.

### Methods

#### Participants

Thirty-six young adults (17 men, age = 20.6 ± 1.16, 18 women, age = 21.1 ± 1.52) and 97 children (40 boys and 50 girls) participated in this study (7.42 ± 0.13). Young adults were recruited via the Subject Pool of the School of Psychology at the University of Lincoln and children were recruited and tested during a ‘Summer Science’ week organised by the School of Psychology (one year from data collection for Experiment 1).

#### Procedure

The procedures for the testing sessions of young adults and children were identical to those used in Experiment 1 with the following minor changes: Each clip was preceded by a sentence indicating the age-group (e.g., “The next clip shows an older adult”), confidence ratings for young adults were not recorded, and the measure for contact was changed from the number of hours per month to eight response categories (adapted from Ebner & Johnson, 2009): less than once per year, once per year, 2/3 times per year, once per month, 2/3 times per month, once per week, 2/3 times per week, more than 3 times per week. The reason for this latter change was that anecdotal reports of participants in Experiment 1 had indicated that that guessing the number of contact hours per month can be difficult.

**Figure 3:**
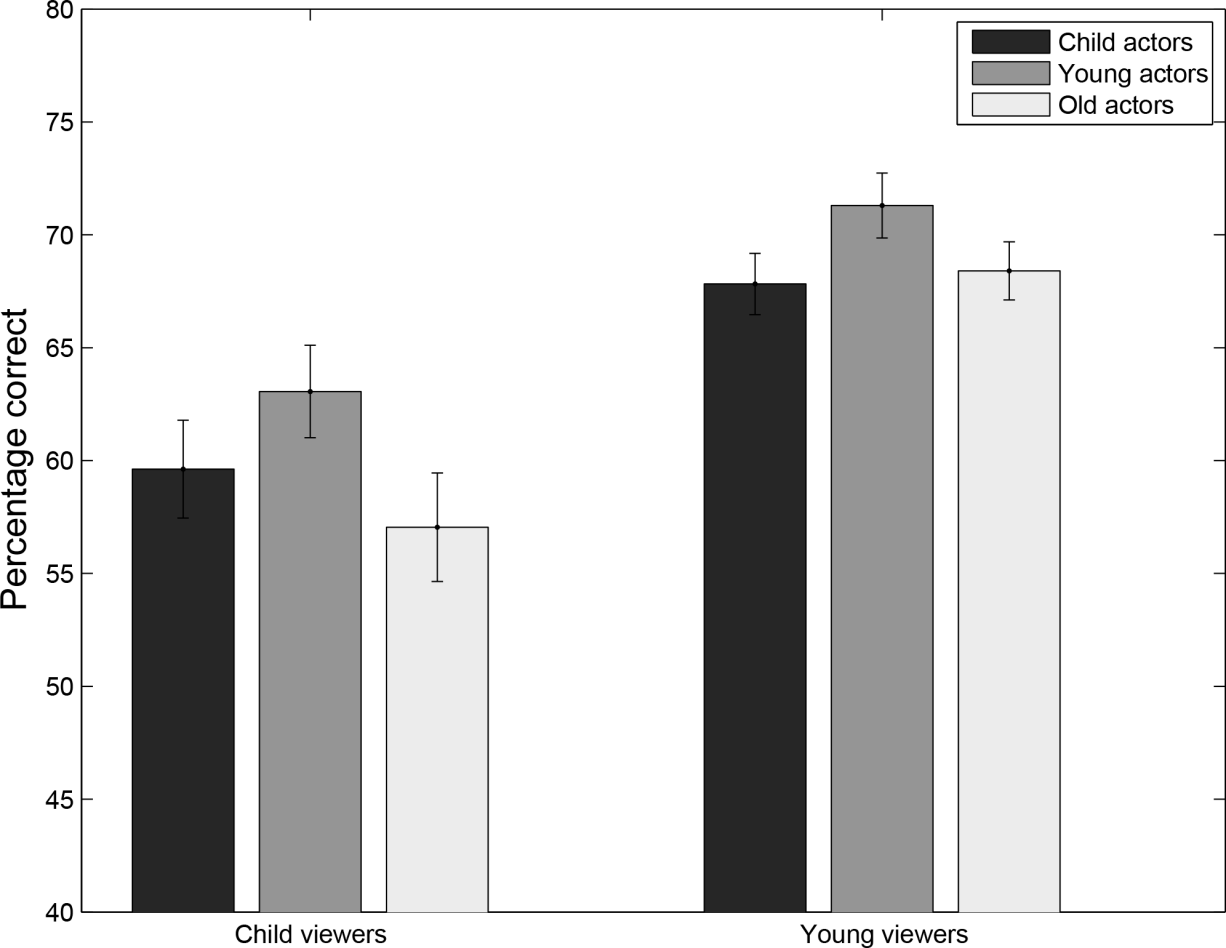
Experiment 2: percentage correct responses for viewers (Young viewers and Child Viewers) as a function of Age-of-Actor (Young actors, Older actors and Children).

### Results

#### Body expression recognition accuracy

Figure 3 provides an overview of the main results of Experiment 2. These results suggest that performance by the younger adults exceeded that of the children and that the highest performance can be found for young actors. To examine the statistical significance of these observations and to incorporate other factors, such the perceived emotion and the gender of the viewer, the same two models as in Experiment 1 were fitted to the data, as shown in  Table 2.

Model 1: Table 2 shows that for experiment 2, in contrast to Experiment 1, the interaction effect between Age-Viewer and Age-Actor was no longer significant for the two models considered. Inclusion of the random slopes did not significantly increase the fit of Model 1(Chi-square (*df* = 2) = 0.95; *p* = 0.62). Comparison of the data plots for Experiment 1 (Fig. 1) and Experiment 2 (Fig. 3) suggests that the overall pattern of results in Experiment 2 is similar to that in Experiment 1, with highest performance for younger actors in both age groups and higher performance by young adult observers. This suggests that adding information about the age of the actor did not influence the results. Such a conclusion is confirmed by an analysis comparing the results of Experiments 1 and 2, testing the effects of the age of the actor, age of the viewer, experiment number and all of the interactions. The three-way interaction was not significant (*z* = 0.16, *p* = 0.87), and neither were any of the two-way interaction (all *p* > 0.33). The age of actor by age of viewer interaction was not significant across both experiments (*p* = 0.335), and only the effect of the age of the viewer was significant (*z* = 2.71, *p* = 0.0067). Post-hoc comparisons for the two age groups separately did not reveal an effect of age of the actors (*both p-values* >  0.70). Post-hoc Bonferroni corrected comparisons (*N* = 3) for each actor age group showed significant differences between young adult viewers and children for young adult actors (*z* = 3.68, *p* < 0.001), older adult actors (*z* = 2.83, *p* = 0.0046), and borderline significant differences for child actors (*z* = 1.94, *p* = 0.053).

**Figure 4:**
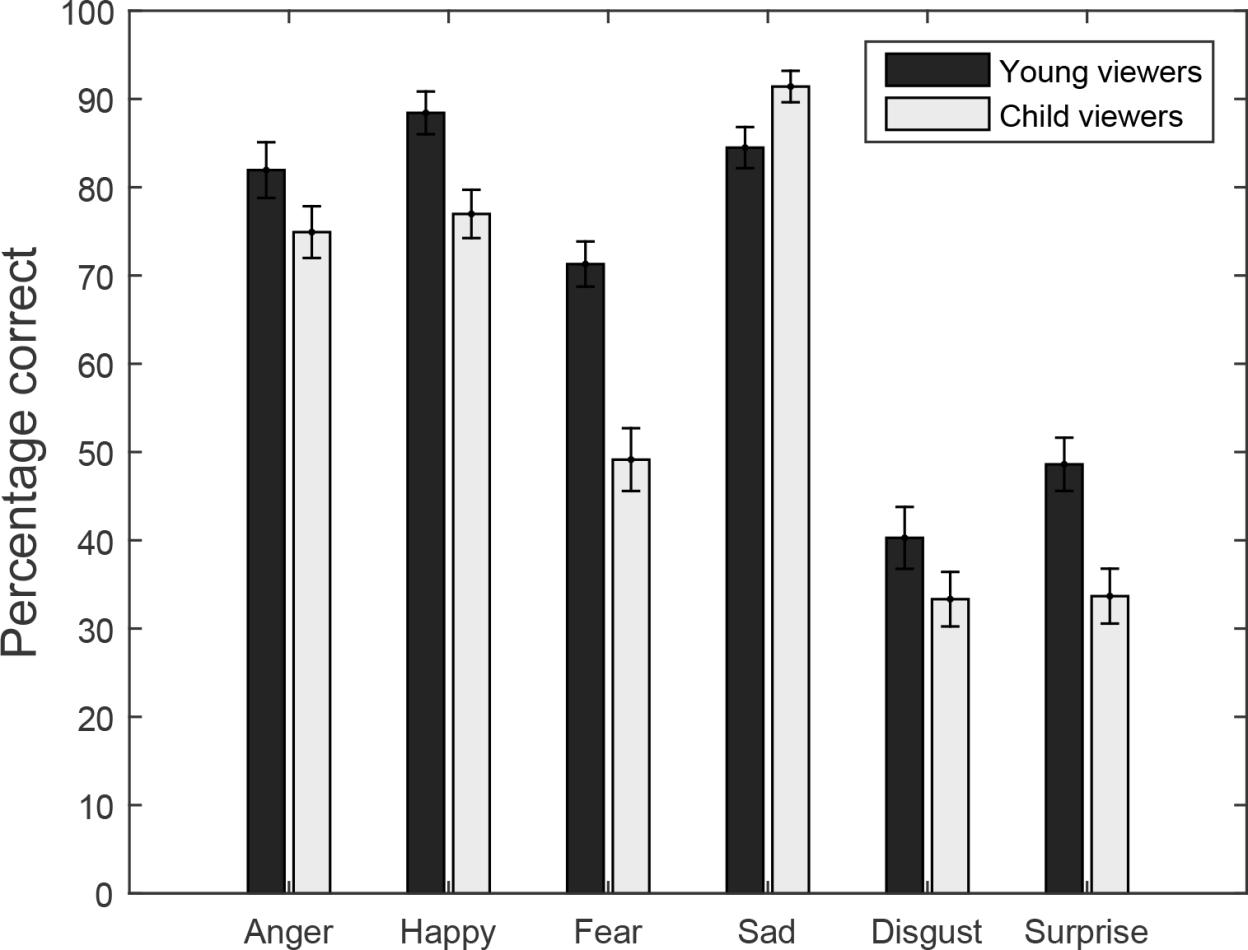
Experiment 2: percentage correct responses for viewers (Young viewers and Child Viewers) as a function of body expression (Anger, Happy, Fear, Sad, Disgust and Surprise).

Model 2: differences between age groups for each emotion are shown in Fig. 4. As in Experiment 1, performance was better for the more basic emotions, but this time children performed lower for Fear emotions. The significant effect of emotion is confirmed in Model 2, as shown in Table 2. Also this pattern of results did not differ from Experiment 1. Testing the interaction with experiment of Model 2 did not reveal any significant interactions and only the effects of emotion (*z* = 4.01, *p* < 0.001) and age-group (*z* = 1.99, *p* = 0.046) remained. Testing the interaction between age of the observer and age of the actor for each emotion separately with Bonferroni corrected comparisons (corrected critical *p*-value = 0.05/6 = 0.0083) revealed significant interactions between actor age and viewer age only for Fear (*p* < 0.001). In a direction comparison, a non-significant three-way interaction between age of the actor, age of the viewer and the experiment (*z* = 0.92, *p* = 0.36) suggests that this interaction for Fear was not influenced by age information about the actors. The results of Experiment 2 therefore replicate those of Experiment 1, and therefore the source of the interaction between age of the actor and the age of the viewer in Experiment 1 must need to be sought in the inclusion of older adults in the first experiment (even though it is the group of children who most clearly violate the own age bias in both experiments).

##### Contact

Initial exploration of potential associations between contact and accuracy were conducted with tests of correlations (Spearman Rho) between estimated contact with people of different age groups (rating 1–8) and categorization accuracy. Ratings for children’s contact with other children were excluded from these analyses as only values 7 (9%) and 8 (90%) were selected. Similar to Experiment 1, only one significant relationship was found: For children only, estimated amount of contact with older adults was significantly related to percentage correct responses for clips of older actors (Rho = 0.38; *p* < 0.001). Moderated Linear Regression was used to investigate if Age-group of the viewer (young adults vs. children) moderates the relationship between contact and percentage correct categorizations of body expressions posed by older adults. Model 1 accounted for a significant amount of variance in children’s ability to recognize body expression posed by older actors (*R*^2^ = 0.*R*^2^, F(2,130) = 8.3, *p* < 0.001). Inclusion of the interaction effect after centring of means resulted in a significant change in *R*^2^ (*R*^2^ change = 0.036, F(1, 129) = 5.5, *p* = 0.02). Analysis of the slopes separately for young adults and children revealed a significant positive slope for children (B = 3.46; t = 4.09, *p* < 0.001) which was not significant for adults (*B* =  − 1.19; *t* = 0.7, *p* = 0.46)

The initial exploratory analysis of correlations between contact and accuracy revealed that the relationship between contact with older adults and accuracy for body expressions of older adults was only significant for younger children (six year old children: Rho = 0.57; *p* = 0.001; seven year old children: Rho = 0.38, *p* = 0.042). To verify if this selective effect for young children could have been due to differences in group sizes, eight, nine and ten year old children were combined for this exploratory correlation analysis to increase the sample size, yet the relationship remained non-significant for the older children. To investigate whether the effects of contact on accuracy is emotion-specific, six and seven year old children (who showed the strongest relationship between contact and accuracy) were divided into two groups: Children with low contact with older adults (*N* = 17) or with more frequent contact with older adults (2–3 times per year or more, *N* = 39). Group-wise comparisons showed that differences between the two groups were most pronounced for angry expressions (*t*(95) = 3.3; *p* = 0.001) and happy expressions (*t*(95) = 2.5; *p* = 0.012).

## General Discussion

The aim of the present study was to investigate the own-age bias and the role of contact in categorizations of emotions from other people’s body movements, shown in the form of point light displays (PLDs), to isolate the movement component from any other possible clues about the other person’s emotion. In the first experiment, no information about the age group of the actors was provided in advance, whereas in a second experiment participants were informed about the age of the actor before the PLD was shown. Comparison of the two experiments showed however, that knowing the age group of the actor did not influence the results. Experiment 1 showed a significant interaction between the observer’s own age and the age of the actor, yet the pattern of results (in both experiments) was not consistent with the predicted pattern of results for own-age biases (where highest performance would be expected for PLDs of actors who belong to the same age-group as the viewer). Figure 1 shows a trend suggestive of a small advantage in accuracy for young adults viewing young adult actors and older adults viewing older adult actors. However, when the analysis only included older and young adult viewers, the interaction effect between viewer’s age and actor’s age was not significant. Likewise, when only older adult viewers and child viewers were included in the analysis, the interaction was again not significant. In addition, the interaction between viewer’s age and actor’s age was not significant when child viewers and young adult viewers were analysed, even when the data of Experiment 1 and Experiment 2 were pooled. The trend for child viewers also seems inconsistent with the the own age bias given that child viewers were somewhat better at categorizing young adult actors than child actors. Moreover, the effect of actor’s age was not significant, neither for young adult viewers, older adult viewers nor for children, in both Experiments. Together, these non-significant results are difficult to reconcile with an age-congruency effect in body expression categorization from PLD. Instead, comparisons between viewer age-groups showed that the interaction observed in Experiment 1 is more likely driven by an advantage for older adult viewers compared to child viewers for PLDs of young adult actors only.

We did, however, find a consistent positive association between the extent of the contact of young children with older adults and their ability to categorize older adults’ expressions. This latter finding suggests that exposure to older adults in early childhood may be important for enhancing recognition of emotions in older adults at that age. Across the two experiments, we also found that basic emotions (fear, sadness, anger and happiness) could be more easily recognized than more complex emotions (disgust and surprise).

There are several possible reasons why the present study did not produce results consistent with an own-age bias in recognition of emotions from PLDs. One possible explanation is that emotion categorization from PLDs of body expressions is not mediated by simulation of the emotions. The role of simulation in emotion expression from facial cues has previously been supported by associations between facial expression categorization and activation of spontaneous facial muscle movements (Dimberg, 1982). Moreover, when participants are prevented from engaging these muscle movements, categorization accuracy of ambiguous facial expression is reduced (Niedenthal et al., 2001; Oberman, Winkielman & Ramachandran, 2007). While an association between emotion recognition and body movements has been observed when participants rate their own mood (Shafir et al., 2013), a similar association between body expression categorization responses and body movements has not been reported yet. However, evidence for commonalities in the mechanisms underlying processing of face and body expressions are accumulating in the literature. Firstly, similar to facial expressions, sensitivity to body expressions develops early and is observable in 6.5 month old infants for fully-lit body expressions (Zieber et al., 2014) and in 8-month-old infants for PLDs of body expressions (Missana, Atkinson & Grossmann, 2015). Secondly, Kret et al. (2013) reported remarkable commonalities in behavioural and physiological measures in response to body and facial expressions of fear, suggesting that threat-related cues from both sources have a similar effect on arousal and attention allocation. Given these similarities in early sensitivity and automatic responses to both facial and body cues, combined with the accumulating evidence for the role of simulation in facial expression categorization (Winkielman et al., 2015; Mitchell & Phillips, 2015), it is difficult to assume that simulation processes would not be engaged for body expressions.

A different explanation for the absence of an own-age bias in the present study is that age-related influences on body movements are rather subtle in PLDs of body expressions compared to the influences of emotions on body movements. Body-expressions are often characterized by emotion-typical movements, such as fist and lower arm shaking for anger, upward arm shaking for happiness, or slow forward folding movement for sadness (De Meijer, 1989; Atkinson et al., 2004; Dael, Mortillaro & Scherer, 2012). Simulation of these emotion-specific movements may be more beneficial for rapid judgements about emotional states in others compared to subtle age-related differences in movement characteristics. In face perception, the own-age bias has been reliably reported for recognition of facial identity (Ebner & Johnson, 2010; He, Ebner & Johnson, 2011; Ebner, He & Johnson, 2011) yet not consistently for expression processing (Ebner, He & Johnson, 2011; Ebner, Johnson & Fischer, 2012; Ebner et al., 2013; Hühnel et al., 2014; Malatesta et al., 1987; Riediger et al., 2011). Whether a similar discrepancy exists between identity and emotion judgements based on PLDs is not yet known and could be explored in future studies. Identity recognition from whole body PLDs has been found to be modest for identification of a friend (e.g., ∼50%) and at chance level for strangers (Loula et al., 2005), but recognition can be increased by pre-training and familiarization to PLDs of strangers (Jacobs, Pinto & Shiffrar, 2004; Troje, Westhof & Lavrov, 2005). Identity and emotion recognition could therefore be directly compared to investigate potential parallels between body and face processing.

In a third explanation more obvious age cues may be required in the body expression clips for influences of age-related movement characteristics to occur. Experiment 2 demonstrates that simply providing verbal information about the age group of the actor did not induce such effects. This may be because age information was not directly relevant for the task at hand, or because age information is evaluated at a later stage of processing. Age cues are more abundant in fully lit whole body expressions and could potentially lead to stronger simulation when viewing videos with own-age actors. Such explanations are in line with evidence for an own age effect on motor resonance in children in the evaluation of grasping hands reaching for graspable objects (Liuzza, Setti & Borghi, 2012).

Interestingly, we found that an increase in contact with older adults led to better categorization of older actors’ expressions in children, particularly in the younger children (aged 6 and 7). These findings support the idea that exposure to expressions of emotions enhances the recognition of those emotions. Our findings also suggest that this exposure effect is strongest for emotions involving rapid movements. The difference between young children with low or high contact with older adults was most pronounced for clips of older actors expressing happy and angry expression, which are both characterized by rapid and jerky movements in young adults (Dael, Mortillaro & Scherer, 2012). A possible reason may be that the reduced velocity in older actors’ movements may have been particularly confusing for the youngest children who have little contact with older adults. Why the association was only observed for younger children is not clear. One possible explanation is that a low contact score on the questionnaire is a more valid measure for the youngest children. These children may not have had the time to build up a solid representation of body movements when contact is low, while older children and adults may have had sufficient time to build up such representations over the years, even if contact is low.

Performance of older adults was significantly lower than for younger adults. This finding is consistent with previous studies, where body expression recognition accuracy was lower in older adults for video clips of fully lit posed body expressions (Ruffman, Ng & Jenkins, 2009; Ruffman, Sullivan & Dittrich, 2009), PLDs of posed body expressions (Insch et al., 2015) and for PLDs of ‘emotional walkers’ (Spencer et al., 2016). Accuracy in body expression recognition was also reduced for child viewers compared to young adults in the present study. Past research suggests that adult levels of body expression categorization can be found for dancing figures and videos of posed body expressions in eight to nine year old children (Boone & Cunningham, 1998; Lagerlöf & Djerf, 2009), whereas a more gradual increase has been observed across childhood and adolescence when PLDs of acted body expressions were used (Ross, Polson & Grosbras, 2012). This gradual increase seems consistent with the developmental trajectory observed in childhood for sensitivity to facial expressions for emotions, such as for fear, disgust and surprise (Gao & Maurer, 2010). Accuracy for disgust and surprise was low for age-groups compared to other four body expressions. Compared to bodily cues, facial expressions for disgust and surprise may be less ambiguous, possibly due to their functional relevance (e.g., constricting nasal passage and closing mouth to reduce inhalation of possible contaminants, Rozin, Lowery & Ebert, 1994; Shariff & Tracy, 2011) and may be more informative for rapid judgements of a person’s emotional state. Both disgust and surprise have not been investigated often with PLDs of body expressions. Only the patch light displays developed by Atkinson et al. (2004) included disgust whereas surprise has not been used so far. While performance for PLDs of disgust was also lowest compared to other expressions in Atkinson et al. (2004), accuracy was higher compared to the present study, which may be due to additional form information from the light emitting strips used in their displays, as argued by Ross, Polson & Grosbras (2012), or due to different levels of variability in actors’ expression of disgust. Greater variability in the enactment of expressions could arguably enhance reliance on retrieval of conceptual and contextual knowledge about a specific emotion (Fugate, 2013; Widen, 2013). Development of conceptual understanding of emotions has been suggested to be of critical importance for expression recognition skills in children (Widen, 2013), which could explain why children performed more poorly on clips for disgust and surprise compared to young adults. In contrast, children’s accuracy for sad expressions was high, even compared to young adults in experiment two. This could be attributable to the early development of sensitivity to sad and happy expression in childhood (Cheal & Rutherford, 2011; Gao & Maurer, 2010), or it may be that sad body expressions were relatively distinctive in the present study, given that most actors included a slow, downward head-movement in the expression of this emotion. Future studies will be required to verify if the age effect for sadness may be specific to the stimulus-set used in the present study.

To conclude, the present study did not find evidence for an own-age bias in body-expression categorization from point light walkers, suggesting that the kinematics of one’s own body movements do not systematically influence the judgment of other’s body expressions. However, contact with older adults correlated with the ability to categorize their expressions by children, suggesting that early exposure to age-specific kinematic information improves young children’s ability to detect emotions in older adults.

## Supplemental Information

10.7717/peerj.2796/supp-1 Supplemental Information 1 Raw data of young and older viewers

10.7717/peerj.2796/supp-2 Supplemental Information 2 Raw data of child viewers

## Additional Information and Declarations

### Data Availability

The following information was supplied regarding data availability: The raw data has been supplied as a Supplemental File.

## References

[ref-1] Ambady N, Rosenthal R (2008). Thin slices of expressive behaviour as predictors of interpersonal consequences: a meta-analysis. Psychological Bulletin.

[ref-2] Atkinson AP, Dittrich W, Gemmell AJ, Young AW (2004). Emotion perception from dynamic and static body expressions in point-light and full-light displays. Perception.

[ref-3] Atkinson AP, Tunstall ML, Dittrich WH (2007). Evidence for distinct contributions of form and motion information to the recognition of emotions from body gestures. Cognition.

[ref-4] Atkinson AP, Vuong QC, Smithson HE (2012). Modulation of the face- and body-selective visual regions by the motion and emotion of point-light face and body stimuli. NeuroImage.

[ref-5] Avieser H, Hassin RR, Ryan J, Grady C, Susskind J, Anderson A, Moscovitch M, Benton S (2008). Angry, disgusted, or afraid? Studies on the malleability of emotion perception. Psychological Science.

[ref-6] Barr DJ, Levy R, Scheepers C, Tily HJ (2013). Random effects structure for confirmatory hypothesis testing: keep it maximal. Journal of Memory and Language.

[ref-7] Barsalou L, Solomon K, Wu L (1999). Perceptual simulation in conceptual tasks.

[ref-8] Bernhardt D, Robinson P (2007). Detecting affect from non-stylised body motions.

[ref-9] Boone RT, Cunningham JG (1998). Children’s decoding of emotion in expressive body movement: the development of cue attunement. Developmental Psychology.

[ref-10] Caligiore D, Pezzulo G, Miall RC, Baldassarre G (2013). The contribution of brain sub-cortical loops in the expression and acquisition of action understanding abilities. Neuroscience and Biobehavioral Reviews.

[ref-11] Castellano G, Villalba SD, Camurri A (2007). Recognising human emotions from body movement and gesture dynamics.

[ref-12] Cheal JL, Rutherford MD (2011). Categorical perception of emotional facial expressions in preschoolers. Journal of Experimental Child Psychology.

[ref-13] Coulson M (2004). Attributing emotion to static body postures: recognition accuracy, confusions and viewpoint dependence. Nonverbal Behavior.

[ref-14] Dael N, Mortillaro M, Scherer KR (2012). Emotion expression in body action and posture. Emotion.

[ref-15] Damasio AR (1999). The feeling of what happens: body and emotion in the making of consciousness.

[ref-16] De Gelder B (2009). Why bodies? Twelve reasons for including bodily expressions in affective neuroscience. Philosophical Transactions of the Royal Society of London. Series B: Biological Sciences.

[ref-17] De Gelder B, Snyder J, Greve D, Gerard G, Hadjikhani N (2004). Fear fosters flight: a mechanism for fear contagion when perceiving emotion expressed by a whole body. Proceedings of the National Academy of Sciences of the United States of America.

[ref-18] De Gelder B, Van den Stock J, Meeren HKM, Sinke CBA, Kret M, Tamiettoa M (2010). Standing up for the body. Recent progress in uncovering the networks involved in the perception of bodies and bodily expressions. Neuroscience and Biobehavioral Reviews.

[ref-19] De Meijer M (1989). The contribution of general features of body movement to the attribution of emotions. Journal of Nonverbal Behavior.

[ref-20] Dimberg U (1982). Facial reactions to facial expressions. Psychophysiology.

[ref-21] Ebner NC, He Y, Johnson MK (2011). Age and emotion affect how we look at a face: visual scan patterns differ for own-age versus other-age emotional faces. Cognition and Emotion.

[ref-22] Ebner NC, Johnson MK (2009). Young and older emotional faces: are there age group differences in expression identification and memory?. Emotion.

[ref-23] Ebner NC, Johnson MK (2010). Age-group differences in interference from young and older emotional faces. Cognition & Emotion.

[ref-24] Ebner NC, Johnson MK, Fischer H (2012). Neural mechanisms of reading facial emotions in young and older adults. Frontiers in Psychology.

[ref-25] Ebner NC, Johnson MR, Rieckmann A, Durbin KA, Johnson MK, Fischer H (2013). Processing own-age vs. other-age faces: neuro-behavioral correlates and effects of emotion. NeuroImage.

[ref-26] Fölster M, Hess U, Werheid K (2014). Facial age affects emotional expression decoding. Frontiers in Psychology.

[ref-27] Fugate JMB (2013). Categorical perception for emotional faces. Emotion Review.

[ref-28] Gallese V (2009). Motor abstraction: a neuroscientific account of how action goals and intentions are mapped and understood. Psychological Research.

[ref-29] Gallese V, Goldman A (1998). Mirror neurons and the mind-reading. Trends in Cognitive Sciencess in Cognitive Sciences.

[ref-30] Gao X, Maurer D (2010). A happy story: developmental changes in children’s sensitivity to facial expressions of varying intensities. Journal of Experimental Child Psychology.

[ref-31] Glowinski D, Dael N, Camurri A, Volpe G, Mortillaro M, Scherer K (2011). Towards a minimal representation of affective gestures. IEEE Transactions on Affective Computing.

[ref-32] Grafton ST, Tipper CM (2013). Decoding intention: a neurergonomic perspective. NeuroImage.

[ref-33] Gunes H, Shan C, Chen S, Tian Y, Komar A, Charkoborty A (2015). Bodily expression for automatic affect recognition. Emotion recognition: a pattern analysis approach.

[ref-34] Harrison V, Hole GJ (2009). Evidence for a contact-based explanation of the own-age bias in face recognition. Psychonomic Bulletin & Review.

[ref-35] Hayes AF (2013). Introduction to mediation, moderation and conditional process analysis. A regression-based approach.

[ref-36] He Y, Ebner NC, Johnson MK (2011). What predicts the own-age bias in face recognition memory. Social Cognition.

[ref-37] Heberlein AS, Atkinson AP (2009). Neuroscientific evidence for simulation and shared substrates in emotion recognition: beyond faces simulation or shared-substrates models of emotion recognition. Emotion Review.

[ref-38] Hühnel I, Fölster M, Werheid K, Hess U (2014). Empathic reactions of younger and older adults: no age related decline in affective responding. Journal of Experimental Social Psychology.

[ref-39] Insch PM, Slessor G, Phillips LH, Atkinson A, Warrington J (2015). The impact of aging and Alzheimer’s disease on decoding emotion cues from bodily motion. Neuroscience.

[ref-40] Jacobs A, Pinto J, Shiffrar (2004). Experience, context, and the visual perception of movement. Journal of Experimental Psychology: Human Perception and Performance.

[ref-41] Jaeger TF (2008). Categorical data analysis: Away from ANOVAs (transformation or not) and towards logit mixed models. Journal of Memory and Language.

[ref-42] Kret ME, De Gelder B (2012). A review on sex differences in processing emotional signals. Neuropsychologia.

[ref-43] Kret ME, Stekelenburg JJ, Roelofs K, De Gelder B (2013). Perception of face and body expressions using electromyography, pupillometry and gaze measures. Frontiers in Psychology.

[ref-44] Lagerlöf I, Djerf M (2009). Children’s understanding of emotion in dance. European Journal of Developmental Psychology.

[ref-45] Liuzza MT, Setti A, Borghi AM (2012). Kids observing other kid’s hands: visuomotor priming in children. Cognition and Consciousness.

[ref-46] Lorey B, Kaletsch M, Pilgramm S, Bischoff M, Kindermann S, Sauerbier I, Stark R, Zendgraf K, Munzert J (2012). Confidence in emotion perception in point-light displays varies with the ability to perceive own emotions. PLoS ONE.

[ref-47] Loula F, Prasad S, Harber K, Shiffrar M (2005). Recognizing people from their movement. Journal of Experimental Psychology: Human Perception and Performance.

[ref-48] Malatesta CZ, Izard CE, Culver C, Nicolich M (1987). Emotion communication skills in young, middle-aged, and older women. Psychology and Aging.

[ref-49] Meeren HKM, Hadjikhani N, Ahlfors SP, Hämäläinen MS, De Gelder B (2008). Early category-specific cortical activation revealed by visual stimulus inversion. PLoS ONE.

[ref-50] Missana M, Atkinson AP, Grossmann T (2015). Tuning the developing brain to emotional body expressions. Developmental Science.

[ref-51] Mitchell RLC, Phillips LH (2015). The overlapping relationship between emotion perception and theory of mind. Neuropsychologia.

[ref-52] Niedenthal P, Barsalou L, Winkielman P, Krauth-Gruber S, Ric F (2005). Embodiment in attitudes, social perception, and emotion. Personality and Social Psychology Review.

[ref-53] Niedenthal PM, Brauer M, Halberstadt JB, Innes-Ker ÅH (2001). When did her smile drop? Facial mimicry and the influences of emotional state on the detection of change in emotional expression. Cognition & Emotion.

[ref-54] Oberman LM, Winkielman P, Ramachandran VS (2007). Face to face: blocking facial mimicry can selectively impair recognition of emotional expressions. Social Neuroscience.

[ref-55] Oosterwijk S, Barrett LF, Shapiro L (2014). Embodiment in the construction of emotion experience and emotion understanding. Routledge handbook of embodied cognition.

[ref-56] Paulus M, Hunnius S, Vissers M, Bekkering H (2011). Imitation in infancy: rational or motor resonance?. Child Development.

[ref-57] Pojednic RM, Clark DJ, Patten C, Reid K, Phillips EM, Fielding RA (2012). The specific contributions of force and velocity to muscle power in older adults. Experimental Gerontology.

[ref-58] Rhodes MG, Anastasi JS (2012). The own-age bias in face recognition: a meta-analytic and theoretical review. Psychological Bulletin.

[ref-59] Riediger M, Voelkle MC, Ebner NC, Lindenberger U (2011). Cognition & Emotion beyond “happy, angry, or sad?”: age-of-poser and age-of-rater effects on multi-dimensional emotion perception. Cognition and Emotion.

[ref-60] Rizzolatti G, Craighero L (2004). The mirror-neuron system. Annual Review of Neuroscience.

[ref-61] Ross PD, Polson L, Grosbras MH (2012). Developmental changes in emotion recognition from full-light and point-light displays of body movement. PLoS ONE.

[ref-62] Rozin P, Lowery, Ebert R (1994). Varieties of disgust faces and the structure of disgust. Journal of Personality and Social Psychology.

[ref-63] Ruffman T, Ng M, Jenkins T (2009). Older adults respond quickly to angry faces despite labeling difficulty. Journals of Gerontology - Series B Psychological Sciences and Social Sciences.

[ref-64] Ruffman T, Sullivan S, Dittrich W (2009). Older adults’ recognition of bodily and auditory expressions of emotion. Psychology and Aging.

[ref-65] Seidler RD, Bernard JA, Burutolu TB, Fling BW, Gordon MT, Gwin JT, Kwak Y, Lipps DB (2010). Motor control and aging: links to age-related brain structural, functional, and biochemical effects. Neuroscience and Biobehavioral Reviews.

[ref-66] Shafir T, Taylor SF, Atkinson AP, Langenecker SA, Zubieta JK (2013). Emotion regulation through execution, observation, and imagery of emotional movements. Brain and Cognition.

[ref-67] Shariff A, Tracy JL (2011). What are emotion expressions for?. Current Directions in Psychological Science.

[ref-68] Spencer JMY, Sekuler AB, Bennett PJ, Giese MA, Pilz KS (2016). Effects of aging on identifying emotions conveyed by point-light walkers. Psychology and Aging.

[ref-69] Troje N, Westhof C, Lavrov M (2005). Person identification from biological motion: effects of structural and kinematic cues. Perception and Psychophysics.

[ref-70] Van de Riet WAC, Grèzes J, De Gelder B (2009). Specific and common brain regions involved in the perception of faces and bodies and the representation of their emotional expressions. Social Neuroscience.

[ref-71] Van der Stock J, Righart R, De Gelder B (2007). Body expressions influence recognition of emotions in the face and voice. Emotion.

[ref-72] Van Elk M, Van Schie HT, Hunnius S, Vesper C, Bekkering H (2008). You’ll never crawl alone: neurophysiological evidence for experience-dependent motor resonance in infancy. NeuroImage.

[ref-73] Vrieze SI (2012). Model selection and psychological theory: a discussion of the differences between the akaike information criterion (AIC) and the bayesian information criterion (BIC). Psychological Methods.

[ref-74] Wallbott HG (1998). Bodily expression of emotion. European Journal of Social Psychology.

[ref-75] Widen SC (2013). Children’s interpretation of facial expressions: the long path from valence-based to specific discrete categories. Emotion Review.

[ref-76] Wiese H, Komes J, Schweinberger SR (2012). Daily-life contact affects te own-age bias and neuroal correltes of face memory in elderly participants. Neuropsychologia.

[ref-77] Wiese H, Wolff N, Steffens MC, Schweinberger SR (2013). How experience shapes memory for faces: an event-related potential study on the own-age bias. Biological Psychology.

[ref-78] Winkielman P, Niedenthal P, Wielgosz J, Eelen J, Kavanagh LC, Mikulincer M, Shaver PR, Borgida E, Bargh J (2015). APA handbook of personality and social psychology, volume 1: attitudes and social cognition.

[ref-79] Zieber N, Kangas A, Hock A, Bhatt RS (2014). Infants’ perception of emotions from body movements. Child Development.

