# Supplementary material for: Age-congruency and contact effects in body expression recognition from point-light displays (PLD)
Source: PeerJ. 2016 Dec 13;4:e2796. doi: 10.7717/peerj.2796 (PMC5157186; doi:10.7717/peerj.2796)
Supplement: Supplemental Information 1 [file peerj-04-2796-s001.pdf]

DATA OF ADULTS

Exp	YA/OA	ppnr	gender	age	ya1fan	ya1fha	ya1ffe	ya1fsd	ya1fdi	ya1fsu	ya2fan	ya2fha	ya2ffe	ya2fsd	ya2fdi	ya2fsu	ya1mar	ya1mha	ya1mfe	ya1msd	ya1mdi
1	1	1	2	18	9	7	6	4	1	6	9	7	6	4	3	3	9	7	6	4	1
1	1	2	2	18	9	7	6	4	7	7	9	7	6	4	4	1	9	7	6	4	7
1	1	3	1	20	9	7	6	4	3	3	9	7	6	4	7	1	9	7	6	4	6
1	1	4	1	18	9	7	6	4	3	3	9	7	1	4	3	1	9	7	7	4	3
1	1	5	2	22	9	7	6	4	3	1	9	9	6	4	9	1	9	4	7	4	6
1	1	6	1	19	9	7	6	4	3	4	9	7	6	4	3	1	9	3	6	4	4
1	1	7	2	18	9	6	6	4	3	4	9	7	6	4	1	1	9	6	6	1	4
1	1	8	2	18	9	3	6	4	3	1	9	7	6	1	3	1	9	9	6	4	1
1	1	9	1	19	9	7	6	6	3	6	9	7	6	3	1	1	9	7	6	4	3
1	1	10	2	21	9	7	6	4	6	6	9	7	6	6	3	1	9	7	4	4	3
1	1	11	1	21	9	7	6	4	3	1	9	7	6	4	4	1	9	7	6	4	1
1	1	12	1	20	9	7	6	4	3	6	9	7	6	4	4	4	9	7	6	4	4
1	1	13	2	20	9	7	6	4	6	6	7	1	6	6	4	1	9	7	6	4	4
1	1	14	1	19	9	7	6	4	3	7	1	6	6	4	4	4	9	9	6	4	4
1	1	15	2	20	9	7	9	4	3	1	9	7	6	4	3	6	9	7	9	4	3
1	1	16	1	20	9	7	6	4	3	7	9	7	6	4	4	1	9	7	6	4	3
1	1	17	1	20	9	7	6	4	3	7	9	9	6	4	4	6	1	7	6	4	4
1	1	18	1	20	9	7	6	4	3	1	9	7	6	7	3	1	9	7	6	4	3
1	1	19	1	20	9	7	6	4	3	1	3	7	6	4	4	6	3	7	6	3	4
1	1	20	2	19	9	7	6	4	3	9	9	7	6	4	4	6	9	7	6	4	4
1	1	21	2	19	9	7	6	4	3	3	9	1	6	4	4	1	9	7	6	4	3
1	1	22	1	19	9	7	6	4	3	3	9	7	6	1	4	6	3	7	9	4	4
1	1	23	1	19	9	7	6	4	3	1	9	7	6	3	4	1	9	7	6	4	4
1	1	24	1	19	9	7	6	4	3	1	9	7	6	4	6	1	9	7	6	4	3
1	1	25	2	19	9	7	6	4	1	1	9	7	6	4	3	1	9	7	6	4	4
1	1	26	2	18	9	9	6	4	3	4	9	7	6	4	3	6	9	7	6	4	3
1	1	27	2	18	9	7	6	4	3	4	9	7	6	3	6	6	9	7	6	4	4
1	1	28	1	19	9	7	6	4	4	1	9	7	6	4	6	1	9	9	9	4	4
1	1	29	2	19	7	7	6	4	3	6	9	6	6	6	6	1	7	1	6	4	6
1	1	30	1	19	9	7	6	4	3	6	9	6	6	6	6	3	9	7	6	4	6
1	1	31	2	21	9	9	6	4	3	1	9	7	6	4	3	1	9	7	6	4	6

1	1	32	2	19	9	7	6	4	4	6	9	7	6	6	3	7	9	1	6	4	3
1	1	33	1	19	9	7	6	4	3	6	9	9	6	4	6	1	9	7	6	4	6
1	1	34	1	21	9	7	6	4	3	6	9	7	6	4	6	1	9	7	6	6	6
1	1	35	2	20	7	7	6	4	3	6	9	7	6	7	3	7	9	7	6	4	6
1	1	36	2	21	9	1	6	4	3	6	9	9	6	4	3	1	9	7	6	4	6
1	1	37	2	19	9	7	6	4	3	1	9	7	6	4	6	1	9	7	6	4	6
1	1	38	2	19	9	7	6	4	4	1	9	1	6	1	6	7	9	7	3	4	6
1	1	39	2	19	9	7	6	4	6	6	9	7	6	4	6	1	9	7	6	4	6
1	1	40	1	19	3	7	6	4	6	6	7	1	6	4	6	7	9	1	6	4	3
1	1	41	2	19	9	7	1	4	3	6	9	7	6	4	3	7	9	7	6	4	6
1	1	42	2	19	9	7	6	4	3	1	9	7	1	3	6	7	7	7	1	4	6
1	1	43	2	19	9	7	6	4	3	1	9	7	6	4	6	1	9	7	6	4	3
1	1	44	2	18	9	7	6	4	3	7	9	7	6	4	3	7	9	7	6	4	3
1	1	45	2	19	9	7	6	4	3	7	9	7	6	4	3	1	9	7	6	6	3
1	1	46	2	19	9	7	6	4	3	7	9	7	6	4	6	9	9	1	6	4	6
1	1	47	2	21	9	7	6	4	3	7	7	7	6	4	7	1	9	7	6	4	6
1	1	48	2	34	9	1	6	4	3	7	9	7	6	3	7	9	9	7	1	4	3
1	1	49	2	18	9	7	6	4	6	1	9	7	6	6	3	1	9	7	6	4	7
1	1	50	2	18	9	7	6	4	3	7	7	7	6	4	7	1	9	7	1	4	7
1	1	51	1	18	9	7	6	4	3	7	9	1	6	6	3	1	7	7	6	4	7
1	1	52	1	22	9	7	6	4	3	7	9	1	6	6	7	1	9	7	1	4	7
1	1	53	1	19	9	7	6	4	3	9	9	1	6	4	9	1	9	7	1	4	3
1	1	54	2	21	9	1	1	4	7	1	9	7	6	4	9	1	9	7	6	4	9
1	1	55	1	18	9	7	6	4	3	9	9	7	6	7	3	1	9	1	6	4	9
1	1	56	2	18	9	7	6	4	9	9	9	7	6	4	9	9	9	7	6	4	9
1	2	1	2	74	9	7	6	4	3	6	9	1	6	3	6	1	9	7	7	4	3
1	2	2	2	75	9	9	6	3	4	7	1	3	6	4	4	3	9	1	6	4	4
1	2	3	2	76	9	7	6	4	3	1	1	7	6	6	3	4	9	7	9	4	1
1	2	4	1	75	9	7	6	1	3	3	9	7	1	3	1	4	9	9	1	4	1
1	2	5	1	67	9	7	6	4	1	3	9	7	6	4	3	6	9	7	1	4	4
1	2	6	1	71	9	7	6	4	3	4	4	7	6	1	3	1	9	3	6	4	3
1	2	7	2	80	9	1	3	4	7	4	6	7	7	3	1	1	9	6	4	9	4
1	2	8	1	68	9	7	6	4	3	1	9	7	6	4	4	6	9	4	6	4	3

1	2	9	1	72	3	3	1	9	9	6	6	6	1	4	4	6	9	3	3	1	4
1	2	10	2	70	9	9	6	4	3	1	3	9	6	4	3	1	9	3	3	4	3
1	2	11	2	73	7	9	7	4	3	1	9	9	6	3	4	1	6	6	6	4	4
1	2	12	2	64	9	7	6	4	3	1	9	7	6	3	4	6	3	7	6	3	4
1	2	13	1	74	9	7	6	4	3	6	3	7	6	3	3	1	1	7	6	4	3
1	2	14	2	67	9	7	6	4	3	6	3	9	9	6	3	6	9	6	1	4	3
1	2	15	2	75	9	1	1	4	9	6	7	1	3	6	4	1	9	9	3	3	4
1	2	16	1	69	9	7	6	4	4	1	9	1	6	4	3	1	9	9	6	4	4
1	2	17	1	74	9	1	6	4	3	6	9	7	6	4	4	1	9	1	6	6	4
1	2	18	2	72	9	1	6	3	4	6	7	7	6	4	4	6	9	1	6	4	6
1	2	19	2	76	9	7	6	4	3	6	7	7	7	6	3	6	9	9	7	4	3
1	2	20	2	81	9	7	6	4	4	6	9	7	6	4	4	1	9	7	6	4	6
1	2	21	1	69	9	7	6	4	3	7	9	7	6	6	6	6	9	7	6	4	6
1	2	22	1	68	9	7	6	4	3	1	9	7	7	9	6	7	7	7	7	4	6
1	2	23	2	76	9	7	6	3	3	7	9	7	1	1	6	7	9	9	7	4	6
1	2	24	1	79	9	7	6	4	4	1	9	7	6	3	3	7	9	1	6	4	3
1	2	25	2	82	9	7	6	4	3	7	9	7	6	3	6	7	7	7	6	6	6
1	2	26	1	86	7	7	6	4	6	1	7	7	6	4	6	7	9	1	6	4	7
1	2	27	2	76	9	7	6	4	3	7	9	7	6	3	6	1	9	7	1	4	7
1	2	28	1	74	9	7	6	4	6	7	9	7	6	3	3	1	9	7	1	4	3
1	2	29	2	79	9	1	6	4	3	7	7	1	6	4	7	1	9	1	1	4	3
1	2	30	2	72	9	7	6	6	6	7	9	1	6	6	7	1	9	7	6	4	3
1	2	31	2	74	9	7	6	4	3	1	9	1	6	6	9	7	9	7	6	4	9
1	2	32	1	76	9	7	6	4	3	1	9	7	1	6	9	7	7	7	1	4	9
1	2	33	1	83	9	7	6	6	3	9	9	7	1	6	9	9	9	1	1	4	9
1	2	34	2	67	9	7	6	4	6	9	9	7	6	9	3	9	9	1	6	4	3
2	1	1	1	20	9	3	7	4	3	3	1	1	6	9	4	1	1	7	6	4	1
2	1	2	1	20	9	7	6	4	3	6	9	7	6	4	3	1	9	7	6	4	3
2	1	3	2	20	9	7	7	4	9	7	9	7	6	4	6	1	9	7	6	4	7
2	1	4	1	19	9	7	6	4	3	6	9	7	6	4	4	1	3	7	6	4	9
2	1	5	1	21	9	7	6	4	3	6	3	7	6	4	9	1	9	7	6	4	4
2	1	6	2	20	9	7	6	4	3	1	3	7	6	4	1	3	9	7	6	4	4
2	1	7	2	21	9	9	6	4	1	7	9	1	6	4	1	4	9	7	6	4	3

2	1	8	2	22	9	7	6	4	3	7	6	9	6	4	1	1	9	7	6	4	6
2	1	9	2	20	9	7	6	4	3	3	9	7	6	1	3	4	9	7	3	4	6
2	1	10	2	20	9	7	6	4	3	3	9	7	6	4	3	1	3	7	6	4	1
2	1	11	1	19	9	7	6	4	3	1	9	7	6	4	3	1	9	3	9	4	1
2	1	12	2	21	9	7	6	4	3	1	9	7	6	3	4	1	9	7	6	4	1
2	1	13	1	20	9	1	1	4	4	4	9	9	6	6	4	6	9	7	6	4	4
2	1	14	2	21	9	7	6	4	3	1	9	7	6	4	3	1	9	7	6	4	3
2	1	15	1	21	9	7	6	4	3	4	9	7	6	6	4	1	9	7	6	4	4
2	1	16	2	19	9	7	6	4	4	6	9	7	7	4	4	6	9	1	7	4	4
2	1	17	1	23	9	9	6	4	3	6	9	7	6	9	3	1	6	7	6	3	3
2	1	18	1	21	9	7	6	4	3	1	9	7	6	4	4	6	9	1	7	4	4
2	1	19	1	21	9	7	6	4	3	1	9	7	6	4	4	1	9	7	6	4	4
2	1	20	1	20	3	7	6	4	3	6	9	3	6	4	3	1	9	7	6	4	4
2	1	21	2	20	9	7	6	4	3	6	9	7	6	4	4	1	9	7	6	9	3
2	1	22	2	20	9	9	6	4	4	6	9	1	6	1	3	6	9	7	6	4	3
2	1	23	2	20	7	1	6	4	3	1	9	7	6	4	4	1	9	7	6	4	4
2	1	24	2	24	9	7	6	4	4	6	9	7	6	4	4	1	9	7	1	4	4
2	1	25	2	22	9	7	6	4	3	6	7	7	6	4	6	1	9	7	6	4	4
2	1	26	1	22	9	7	6	4	3	1	9	7	6	4	6	1	9	7	6	6	6
2	1	27	2	22	9	1	6	6	6	7	9	1	6	3	6	1	9	7	6	4	6
2	1	28	1	20	9	7	6	4	3	7	9	7	6	4	6	6	9	7	6	4	6
2	1	29	2	21	9	7	6	4	3	1	9	7	6	4	6	1	9	7	6	4	3
2	1	30	1	20	9	7	6	4	3	7	7	9	6	4	3	7	9	9	1	4	6
2	1	31	2	21	9	7	6	4	3	7	9	7	6	6	6	1	9	9	1	4	6
2	1	32	1	20	9	7	6	4	3	1	9	7	6	6	3	7	7	7	6	4	3
2	1	33	2	25	9	7	6	4	3	7	9	7	6	4	7	1	7	7	6	4	6
2	1	34	1	19	7	7	1	4	6	1	7	7	1	9	9	7	7	7	6	4	7
2	1	35	1	22	9	7	6	4	6	7	9	7	6	4	3	7	9	7	6	6	9
2	1	36	1	22	9	7	6	4	3	9	9	9	6	4	9	9	9	7	6	4	9

Variable names categorization task Experiments 1 and 2:

Example: ‘ya1fan’: ‘ya’= young adult actor, ‘1f’ = female one, ‘an’ = angry

‘ya’, ‘ch’, ‘oa’ = young adult actor, child actor, older adult actor
‘1f’, ‘2f’, ‘1m’, ‘2m’ = female 1, female 2, male 1, male 2
‘an’, ‘ha’, ‘fe’, ‘sd’, ‘di’, ‘su’ = angry, happy, fearful, sadness, disgust, surprise

Response codes:
9=angry
7=happy
6=fear
4=sad
3=disgust
1=surprise

Experiment 1, measure for contact:
‘hpm_OA’ = estimated average number of hours per month spent with older adults
‘hpm_YA’ = estimated average number of hours per month spent with young adults
‘hpm_CH’ = estimated average number of hours per month spent with other children

Note: Answers that were given in other temporal units (e.g. ‘once every three months’) were
Recalculated to estimated number of hours per month.

Experiment 2, measure of contact:
‘Cont_OA’: Rating for contact with older adults
‘Cont_YA’: Rating for contact with young adults
‘Cont_CH’: Rating for contact with children

ya1msu	ya2mar	ya2mha	ya2mfe	ya2msd	ya2mdi	ya2msu	ch1fan	ch1fha	ch1ffe	ch1fsd	ch1fdi	ch1fsu	ch2fan	ch2fha	ch2ffe	ch2fsd	ch2fdi	ch2fsu	ch1mar	ch1mha	ch1mfe
1	9	7	6	4	1	1	9	7	6	4	1	7	1	7	6	4	4	1	1	7	6
9	9	1	3	4	1	3	9	7	6	6	4	9	9	7	9	1	7	3	9	7	4
1	1	7	6	4	1	1	9	7	6	4	6	1	9	7	6	4	6	1	9	7	6
1	3	7	6	4	3	1	9	7	6	1	3	3	9	6	6	4	3	1	9	7	6
1	9	7	6	4	4	3	9	7	6	4	1	3	9	7	6	4	4	1	9	7	6
1	9	7	7	4	4	9	9	7	4	3	3	4	9	7	6	4	4	1	1	7	7
4	9	3	9	4	3	1	9	7	3	3	1	1	9	9	1	4	1	3	9	7	6
3	9	6	6	4	4	9	9	7	6	6	4	4	9	7	6	4	1	1	9	7	6
1	3	7	6	4	4	4	3	7	6	6	3	1	9	7	6	4	4	1	3	7	9
6	9	7	6	4	4	1	9	7	6	4	4	6	9	7	4	4	4	4	3	7	6
6	9	7	6	4	3	4	9	7	7	4	3	6	3	7	3	4	4	4	9	7	7
6	9	7	6	4	3	7	9	7	6	4	3	6	9	3	6	4	4	6	9	7	6
7	9	7	6	4	4	1	9	7	6	6	4	6	9	7	6	4	4	1	9	7	6
7	9	7	6	4	4	1	9	7	6	4	3	7	9	7	3	4	3	1	3	7	6
7	3	7	6	4	4	7	9	7	6	4	4	7	9	7	6	4	3	6	9	7	6
1	9	7	6	4	4	1	9	7	6	4	4	7	9	7	7	4	4	1	9	7	6
1	3	7	6	4	4	7	9	7	6	4	3	1	9	7	6	4	3	6	9	7	6
3	9	7	6	7	4	7	9	7	6	4	4	1	9	7	6	4	4	1	3	7	9
3	9	7	1	4	3	1	7	7	6	4	4	1	9	7	6	4	4	6	9	7	1
4	3	7	6	4	3	1	9	6	6	4	4	1	9	7	6	4	4	1	3	7	6
1	9	7	6	4	3	1	9	7	6	4	4	1	4	7	6	4	4	1	9	7	6
4	3	7	6	4	6	1	9	7	6	4	4	7	9	7	6	4	4	6	3	7	6
9	4	7	1	4	6	1	9	7	7	4	4	3	9	7	7	4	6	1	4	7	1
9	9	7	6	4	3	1	9	7	6	4	3	3	9	7	7	4	6	6	4	7	1
1	4	7	6	4	6	1	9	7	6	4	6	4	9	7	6	4	6	1	4	7	4
9	9	9	6	4	3	6	9	7	7	4	6	1	9	3	6	4	3	6	9	7	3
1	9	7	6	4	3	6	9	7	6	7	6	4	9	9	9	4	6	6	6	7	3
1	9	7	6	4	6	1	9	7	6	4	6	1	9	7	9	4	6	1	9	7	6
1	6	7	6	4	6	1	9	7	6	4	3	6	9	7	6	4	6	1	6	7	6
1	9	7	6	4	6	1	9	3	6	4	3	6	9	9	6	4	3	1	6	7	6
7	9	9	6	4	6	6	7	7	6	1	3	6	6	7	9	4	3	1	9	7	6

1	9	7	6	4	6	6	9	7	9	3	6	6	9	7	6	4	6	7	7	7	6
7	9	7	6	4	3	6	9	7	6	4	6	1	9	7	1	4	3	1	7	7	6
7	6	1	6	4	3	1	9	7	9	4	3	6	9	7	1	4	6	7	9	7	6
7	1	7	6	4	6	7	9	9	6	4	3	1	9	7	6	3	3	7	9	7	6
7	9	1	6	4	3	1	9	7	6	4	6	6	9	1	6	4	3	1	9	7	6
7	9	7	6	4	6	1	9	7	9	3	6	1	9	7	1	4	6	1	7	7	7
1	9	7	6	4	6	1	9	7	6	4	6	6	9	7	1	4	3	7	7	7	7
7	9	7	6	4	6	1	9	7	6	4	6	6	9	7	6	4	6	1	9	7	7
1	7	7	6	4	6	7	7	9	1	6	6	6	7	7	6	6	6	1	9	7	6
7	7	7	1	1	3	1	9	7	6	4	3	6	9	7	6	4	3	7	9	7	9
6	9	7	6	4	3	7	9	7	1	6	6	7	7	7	6	4	3	7	9	7	6
6	9	7	6	4	6	7	9	7	1	4	6	7	9	7	1	4	6	7	7	7	9
6	7	7	1	3	7	1	9	7	6	4	6	7	9	7	6	4	6	1	9	7	9
6	9	7	6	6	7	6	9	7	1	4	4	7	9	7	6	4	3	7	7	9	6
6	9	7	6	4	7	1	9	7	6	4	3	1	9	7	6	4	6	9	9	1	6
1	7	7	6	4	7	6	9	7	6	4	7	7	9	7	6	4	7	1	7	7	1
1	9	7	6	6	3	1	9	1	6	6	3	7	9	1	6	6	3	9	9	7	6
6	9	7	6	4	9	1	9	7	1	7	7	7	9	1	6	4	7	9	9	7	6
6	9	7	6	4	3	1	9	1	6	4	7	7	9	7	6	4	7	1	7	7	1
1	7	7	6	4	3	6	9	1	6	4	7	1	9	7	1	4	3	1	9	7	1
6	7	1	6	4	3	6	9	7	6	4	3	1	9	7	6	4	7	1	7	7	1
6	9	7	6	4	3	6	9	7	1	4	3	9	9	7	6	4	3	1	9	7	1
6	9	7	6	4	9	1	9	7	6	4	9	1	9	7	6	4	9	1	7	7	6
6	7	7	6	4	3	1	9	1	6	4	9	9	9	1	6	4	9	1	7	7	1
1	9	7	6	4	9	1	9	7	6	4	9	9	7	7	6	4	9	1	7	7	6
1	7	7	6	3	1	6	4	6	6	3	3	3	9	7	6	4	1	1	3	7	4
7	9	3	6	4	1	6	9	7	6	3	4	1	9	7	6	4	3	1	7	7	3
1	9	7	6	3	3	1	9	7	4	4	1	4	9	7	6	4	3	7	9	7	9
1	9	7	4	4	4	1	9	7	3	6	1	6	9	7	4	4	1	3	9	7	7
7	9	7	3	4	4	7	9	7	6	4	3	7	9	7	6	4	3	1	7	7	7
7	9	7	6	4	3	1	6	7	6	6	3	3	9	7	6	1	3	1	9	7	6
7	7	7	3	6	4	7	9	3	6	9	4	1	9	7	9	4	3	1	4	7	4
1	7	3	6	4	4	9	1	7	3	4	3	1	9	7	6	4	3	1	9	7	4

7	7	9	9	4	4	9	3	9	6	1	4	3	7	3	9	4	1	3	1	9	6
7	7	4	6	4	4	1	9	7	6	4	4	6	9	7	6	3	3	4	9	7	6
1	3	7	9	4	3	1	9	7	9	3	3	6	9	7	3	4	4	1	9	7	1
7	3	1	6	4	3	3	3	7	6	4	4	6	3	7	6	3	3	4	9	7	6
6	9	1	6	4	4	3	9	7	6	3	3	1	9	7	3	4	4	6	1	7	6
6	3	7	7	4	3	4	9	9	9	3	4	1	9	9	6	4	4	6	6	7	4
6	1	7	7	6	4	4	9	1	6	3	4	1	9	7	7	4	3	1	6	7	3
1	1	7	7	4	6	7	9	7	7	6	3	6	9	7	6	4	4	1	9	7	3
6	9	6	1	4	3	1	9	7	7	6	3	1	6	9	7	3	4	6	9	7	6
6	9	9	6	4	6	7	7	7	6	6	4	1	9	7	6	4	3	1	9	7	9
6	6	7	1	4	3	6	9	1	7	6	4	1	9	1	6	4	4	6	3	7	9
6	9	9	1	4	6	6	9	7	6	9	6	6	9	7	7	6	4	6	9	7	7
1	6	7	6	4	3	6	9	7	6	1	3	6	9	1	1	4	4	6	3	7	7
4	9	7	6	4	3	6	9	7	7	3	3	6	9	7	6	4	6	6	9	7	6
1	9	7	1	4	6	1	9	7	1	3	6	6	9	7	1	4	6	7	9	7	7
1	9	7	6	4	6	7	9	7	1	3	6	7	9	7	6	4	6	7	3	7	6
4	4	1	6	4	3	7	7	7	6	4	6	7	9	7	6	6	3	7	9	7	6
1	7	7	6	4	6	7	7	1	1	3	6	7	9	7	6	6	6	1	9	7	6
9	7	1	6	4	3	7	9	7	6	4	3	1	9	7	6	4	6	1	7	7	1
9	7	7	6	4	7	1	7	7	6	4	6	7	9	7	1	4	6	7	7	7	3
3	3	1	6	4	7	1	9	7	6	4	3	7	7	7	6	4	3	7	9	7	6
1	1	7	6	4	9	6	7	7	6	6	7	1	9	7	6	9	7	1	7	7	7
1	6	7	6	4	3	6	9	7	6	6	7	7	7	1	1	4	7	7	7	7	9
3	9	7	6	4	3	6	9	7	1	6	9	7	9	7	6	4	9	7	9	7	6
1	9	7	1	4	9	1	9	7	1	6	9	9	9	7	1	4	9	9	9	7	9
1	9	7	6	4	9	6	9	7	1	9	9	9	9	7	6	4	9	9	7	7	6
7	7	1	7	3	4	6	7	3	6	3	4	3	9	1	9	4	4	1	7	9	1
1	9	7	6	4	3	1	9	7	6	6	1	4	9	7	1	4	9	1	1	7	6
6	1	7	6	4	1	1	9	7	6	4	3	6	9	7	6	4	6	3	3	7	7
9	7	7	6	4	3	1	9	7	6	3	3	7	9	7	7	4	4	1	3	7	9
3	9	7	7	4	1	1	9	7	6	4	1	1	9	7	6	4	3	1	6	7	6
1	1	7	6	4	3	1	9	7	6	4	3	1	9	7	4	4	3	1	7	7	6
1	3	9	6	4	1	6	9	1	6	4	1	1	1	1	6	4	4	3	9	7	1

3	9	7	9	4	4	1	9	7	6	4	3	1	9	7	6	4	1	1	7	7	6
1	9	7	6	4	4	6	9	7	6	4	4	9	9	7	6	4	3	1	7	7	6
7	9	7	6	4	3	6	9	7	6	4	4	3	9	7	6	4	1	1	9	7	6
1	9	7	6	4	3	1	9	7	6	4	4	1	9	7	6	4	1	4	9	7	4
4	9	7	6	4	4	6	9	1	6	4	3	3	3	7	6	4	4	1	9	7	3
4	3	9	6	4	4	1	9	7	6	4	4	4	9	7	3	4	4	4	1	7	9
9	9	7	1	4	3	1	9	7	6	4	4	1	9	7	9	4	3	6	3	7	6
1	9	7	6	4	3	1	9	7	6	4	3	4	3	7	6	4	3	1	9	7	6
1	9	7	6	4	4	1	9	7	9	4	4	6	9	7	6	4	4	6	9	7	9
6	9	7	6	4	4	7	9	7	6	1	4	6	9	7	6	4	4	1	3	3	6
1	6	7	6	4	4	7	9	7	6	4	4	6	9	7	6	4	4	1	9	7	6
6	9	7	6	4	4	1	9	7	6	4	3	6	9	7	9	4	4	6	6	7	7
1	9	7	6	4	3	1	9	7	6	3	3	1	9	7	7	6	3	6	9	7	7
6	9	7	6	9	3	7	9	7	6	4	3	6	9	7	7	4	3	1	9	7	6
1	7	7	6	4	4	1	9	7	6	4	4	6	9	7	7	4	4	1	9	1	6
1	7	7	6	4	6	1	9	7	6	4	6	1	9	7	6	4	4	6	9	7	7
7	9	7	6	4	6	1	9	7	6	4	6	6	9	7	6	4	3	6	9	7	6
1	9	7	6	4	3	1	9	7	6	4	6	1	9	7	1	4	4	7	9	7	6
7	9	7	6	6	6	1	9	7	7	4	3	7	9	7	6	4	3	1	7	7	6
7	9	7	6	4	6	7	9	7	6	4	3	1	9	7	6	4	6	1	9	7	1
1	9	7	6	4	3	7	9	7	6	6	6	7	9	7	6	4	6	7	9	7	6
1	9	7	6	4	3	9	9	9	6	4	6	7	7	7	6	4	6	1	7	1	6
6	7	7	6	4	6	3	9	7	6	4	6	7	9	7	6	4	3	1	9	7	6
6	9	7	6	4	6	1	9	7	6	4	7	7	9	7	6	4	6	7	9	7	6
1	9	7	6	4	3	1	9	7	6	4	9	7	9	7	1	4	6	7	9	7	1
6	9	7	6	4	1	1	9	7	6	4	3	9	9	7	1	4	6	7	9	7	1
7	0	7	1	4	9	3	7	9	1	4	9	1	9	9	1	4	7	1	7	9	6
7	9	7	6	4	9	4	9	7	6	6	4	7	7	9	6	4	9	7	9	7	1
7	9	7	1	4	3	4	9	7	1	9	6	6	7	7	6	4	9	9	9	9	6

ch1msd	ch1mdi	ch1msu	ch2mar	ch2mha	ch2mfe	ch2msd	ch2mdi	ch2msu	oa1fan	oa1fha	oa1ffe	oa1fsd	oa1fdi	oa1fsu	oa2fan	oa2fha	oa2ffe	oa2fsd	oa2fdi	oa2fsu	oa1mar
4	4	1	9	7	6	4	1	1	9	7	4	4	1	1	9	7	6	4	4	1	9
4	1	9	1	7	6	6	1	9	4	6	6	1	4	7	9	6	6	4	1	1	9
4	1	1	9	7	1	4	4	7	9	7	3	4	3	1	9	7	6	4	1	1	9
4	3	1	9	7	6	4	3	1	9	7	6	4	4	3	9	7	6	4	6	1	9
4	4	1	9	7	1	4	4	1	1	7	3	4	6	1	9	7	6	4	3	1	9
3	4	1	6	7	1	4	4	7	9	7	7	4	3	3	7	3	3	4	6	3	9
4	4	4	9	7	6	4	4	1	9	7	7	4	6	1	4	7	6	7	4	3	9
4	4	3	9	7	6	4	4	7	9	7	7	4	3	1	9	7	6	4	4	1	9
4	3	1	9	7	1	1	4	7	3	7	6	3	3	4	9	7	6	4	1	4	9
4	4	6	9	7	6	4	4	7	9	7	6	4	3	1	1	7	6	1	1	4	9
4	4	6	9	7	1	4	3	1	9	3	9	4	1	1	9	7	6	4	4	1	9
4	3	6	9	7	6	4	3	6	9	7	6	4	1	4	4	7	7	4	4	6	9
4	4	7	9	7	6	3	4	1	9	7	6	4	4	6	9	7	6	4	4	1	9
4	4	7	9	7	6	4	4	1	9	7	9	4	4	1	9	9	6	4	4	1	9
4	3	7	9	7	6	4	4	6	9	7	6	4	4	6	9	7	9	4	3	6	9
4	3	1	3	7	1	4	3	1	3	9	9	6	4	1	9	7	6	4	4	1	9
4	4	1	9	7	6	4	6	1	9	9	6	4	4	6	9	7	6	4	4	1	9
4	3	3	9	7	1	4	3	1	9	7	6	4	4	1	9	7	6	4	4	1	9
4	3	3	9	7	9	4	3	1	9	7	1	4	3	1	9	7	6	4	4	6	1
4	3	4	9	7	9	4	3	1	9	7	6	4	3	1	9	9	6	4	4	6	9
4	6	1	9	7	9	3	3	6	9	7	6	4	3	1	9	7	6	4	3	7	9
4	6	4	9	7	7	4	3	1	4	7	6	4	4	1	9	7	6	4	3	1	9
4	3	9	9	7	6	4	3	1	9	7	1	4	4	1	9	7	6	4	4	1	9
4	3	9	9	7	7	4	3	6	9	7	6	4	4	1	9	7	6	3	4	1	9
4	6	1	9	7	7	4	6	1	9	7	1	4	3	7	6	7	6	4	6	1	3
4	6	9	9	7	3	4	3	1	9	7	1	4	3	7	3	7	6	4	6	1	9
4	6	1	9	7	3	4	3	1	6	7	1	4	4	1	9	7	6	4	3	7	3
4	6	1	9	7	4	4	6	1	9	7	1	4	4	1	3	7	6	4	6	1	6
4	3	1	9	7	6	4	3	1	9	7	6	4	6	7	9	1	6	4	6	7	9
4	6	1	9	7	6	4	3	6	9	7	7	4	6	1	9	7	6	6	3	1	9
4	3	7	9	7	6	4	6	1	9	7	9	4	3	1	9	1	6	4	3	7	9

4	6	1	9	7	1	4	3	6	9	7	1	4	3	7	9	1	9	4	6	6	9
4	6	7	9	7	6	4	3	1	7	7	4	4	6	1	9	7	6	4	3	1	9
4	6	7	9	7	6	4	3	1	9	1	3	4	6	9	7	7	6	4	6	6	9
4	3	7	9	7	6	4	6	6	9	7	6	4	6	1	9	7	1	4	6	6	9
6	3	7	9	7	6	4	3	1	9	7	6	4	3	1	9	7	6	4	3	1	9
4	6	7	9	7	6	4	6	1	9	7	3	4	3	1	9	7	6	4	3	6	9
4	3	1	9	7	1	4	6	1	9	7	7	4	6	1	9	7	6	4	6	1	9
4	3	7	9	7	6	4	6	1	9	7	7	4	6	1	9	7	6	4	6	1	9
4	3	1	9	7	6	4	6	1	7	1	7	4	6	1	9	7	6	4	6	1	9
4	3	7	9	7	6	4	6	6	9	7	6	4	6	9	9	7	6	4	6	9	9
4	3	6	9	7	1	4	7	3	9	7	9	4	6	1	9	7	1	4	6	9	7
4	6	6	9	7	9	4	7	1	9	7	9	4	6	1	7	7	6	4	6	1	9
4	7	6	9	7	6	6	3	1	9	7	9	4	6	1	9	1	1	4	6	1	9
4	3	6	9	7	6	6	3	1	9	1	6	4	6	1	9	7	6	4	3	7	9
4	3	6	9	7	9	4	3	4	9	7	6	4	6	1	7	7	6	4	3	7	9
4	3	1	9	7	6	6	7	1	9	7	6	4	7	1	9	7	6	4	3	7	9
4	3	1	9	7	6	4	3	4	9	7	1	4	7	1	9	7	6	4	3	1	9
4	7	6	7	7	6	4	7	1	9	7	6	4	7	1	9	7	6	4	7	1	9
4	3	6	9	7	7	4	3	1	9	7	6	4	3	1	9	7	6	4	3	7	7
4	7	1	9	7	6	4	3	1	9	7	1	4	7	1	9	7	6	4	7	1	9
4	7	6	9	7	7	4	9	1	7	1	1	4	9	1	7	7	1	6	7	1	9
4	9	6	7	7	6	7	3	1	7	7	1	6	9	1	9	7	6	4	7	1	9
4	3	6	9	7	3	4	9	3	9	7	1	4	3	1	9	7	6	4	9	1	9
4	9	6	9	7	4	4	3	1	9	7	1	4	3	1	9	7	6	4	9	7	7
4	9	1	9	7	6	4	9	1	9	7	6	4	9	1	9	7	6	4	9	1	9
4	3	1	9	7	1	4	1	1	9	3	4	4	4	1	9	1	6	4	3	7	9
4	3	7	9	9	6	4	1	7	7	3	6	4	3	3	9	3	6	4	3	6	7
4	3	1	9	7	6	4	3	1	9	6	3	4	1	3	9	7	6	3	1	1	9
4	3	1	9	7	6	4	3	1	7	7	3	4	1	1	9	7	4	4	1	1	1
4	1	7	9	7	1	4	4	1	7	6	9	4	4	1	9	7	6	4	4	6	9
4	3	7	9	7	1	6	3	1	9	7	7	4	3	1	9	7	6	4	4	1	9
4	4	7	1	7	1	1	4	7	7	7	7	4	4	1	9	6	3	3	4	3	9
4	4	1	9	7	1	3	3	7	7	7	6	4	4	1	9	7	3	4	3	1	9

4	3	7	9	1	7	3	3	7	3	9	1	4	4	1	1	7	9	3	4	3	3
4	4	7	3	7	7	4	3	1	3	7	6	4	4	1	9	7	6	4	3	4	3
4	3	1	9	7	6	3	3	1	9	7	6	4	3	1	9	9	6	4	3	1	3
4	4	7	9	7	6	4	3	1	3	7	6	4	4	1	6	9	6	3	4	1	9
4	4	6	9	7	6	3	3	7	9	7	6	4	3	1	9	7	6	4	3	4	9
4	4	6	9	7	6	6	4	1	9	7	1	4	4	1	9	7	6	4	3	6	9
4	6	6	3	7	7	6	4	6	6	9	1	4	4	1	9	9	9	6	4	6	9
6	6	1	7	7	9	4	4	6	9	7	4	4	3	6	3	9	7	4	4	6	9
4	6	6	9	7	6	4	4	1	9	9	3	4	6	1	3	7	6	6	4	6	9
4	3	6	9	1	6	4	6	6	6	7	6	4	3	6	9	1	6	4	3	6	4
4	3	6	9	7	6	6	6	1	9	7	3	4	6	1	9	7	6	4	3	6	9
4	6	6	7	7	6	6	6	6	1	7	6	6	6	7	9	1	6	4	6	1	9
4	6	1	9	7	9	4	3	1	1	9	9	4	6	1	9	7	6	4	3	9	6
4	3	4	9	7	3	4	6	6	9	7	9	4	6	1	9	7	7	4	6	9	6
4	3	1	9	7	6	4	3	1	4	7	7	4	6	1	9	7	7	4	6	1	7
4	6	1	9	7	3	4	3	9	9	7	7	4	3	7	9	1	1	4	6	7	9
4	3	4	9	7	6	4	3	1	7	7	6	4	7	1	7	1	6	6	6	1	9
4	7	1	9	7	6	4	6	1	7	7	7	4	7	1	9	7	6	4	6	1	9
4	7	9	9	7	4	4	7	1	7	1	1	4	9	1	7	7	1	1	6	1	9
4	9	9	9	7	6	4	7	4	9	7	6	4	3	1	7	7	6	9	7	7	9
4	3	3	7	7	9	4	3	1	3	7	6	4	9	1	9	7	6	4	3	7	7
4	3	1	9	7	9	9	9	3	1	7	6	3	3	9	9	7	6	4	7	7	9
4	9	1	9	7	7	4	3	1	6	1	1	4	9	1	9	7	6	4	3	7	7
4	3	3	9	7	3	4	9	4	9	1	1	4	6	1	9	7	1	4	9	7	7
4	3	1	9	7	6	4	3	1	7	7	1	4	4	1	9	7	1	4	9	1	7
4	9	1	9	7	7	4	3	3	9	1	1	4	3	9	9	1	1	4	9	7	9
4	1	7	1	7	3	4	1	7	1	7	6	4	1	3	1	1	9	1	4	7	9
4	4	1	9	7	9	4	3	1	9	7	6	4	6	1	9	7	6	4	3	7	9
4	3	6	9	7	7	4	3	9	9	7	1	4	3	4	9	7	6	4	1	1	9
4	3	9	9	7	7	4	3	1	7	7	1	4	4	1	3	7	6	4	3	1	9
4	4	3	9	7	6	4	1	1	9	7	1	4	4	6	9	7	6	4	1	6	9
4	4	1	9	7	6	4	4	1	1	7	6	4	1	6	9	7	6	4	3	6	9
4	3	1	9	7	6	4	3	7	9	7	1	4	1	1	9	7	6	4	1	3	9

4	4	3	9	7	6	4	4	1	9	7	7	4	3	6	9	7	9	4	4	3	9
4	3	1	9	7	6	4	3	1	9	7	7	4	3	1	9	7	6	4	3	1	9
4	4	7	9	7	1	4	4	1	9	7	6	4	3	1	9	9	6	4	4	1	1
4	3	1	9	7	1	4	3	7	9	7	6	4	3	1	3	7	6	3	4	1	9
4	3	4	9	7	1	4	4	7	9	7	6	4	1	6	9	7	7	4	4	1	9
4	4	4	7	9	6	4	3	6	3	9	7	4	4	1	9	7	6	4	4	1	9
4	3	9	9	7	6	1	3	1	3	7	9	4	4	1	9	7	6	4	4	1	9
4	3	1	9	7	3	4	3	1	9	7	9	4	3	1	9	7	6	4	3	4	9
4	4	1	9	7	6	4	3	6	9	7	3	4	3	7	9	7	6	4	3	4	9
4	3	6	9	9	6	4	3	1	9	7	4	4	3	1	6	7	6	6	4	1	7
4	4	1	9	7	6	4	4	1	9	7	1	4	3	1	9	7	6	4	4	6	9
6	4	6	9	7	4	4	4	6	9	7	1	4	4	1	9	7	6	4	3	1	9
4	6	1	9	7	9	3	4	1	6	7	6	4	4	1	9	7	6	4	3	1	9
4	6	6	9	7	9	4	4	1	9	7	1	4	4	7	9	7	7	4	3	6	9
4	3	1	9	7	7	4	4	1	9	7	6	4	4	1	9	7	6	4	3	1	9
4	3	1	9	7	7	9	6	4	9	7	1	4	4	1	9	7	6	4	4	6	9
4	6	7	9	7	6	4	6	1	9	7	7	4	4	1	9	7	6	6	6	1	9
4	3	1	9	7	7	4	3	1	9	7	6	4	4	1	7	7	1	4	3	1	9
4	6	7	9	7	6	4	3	1	9	7	6	4	3	1	9	7	6	4	6	6	9
4	6	7	9	7	6	4	6	1	9	7	6	4	6	7	7	7	1	4	6	7	9
4	3	1	9	7	6	4	6	1	9	7	7	4	6	1	9	7	6	4	6	1	9
4	6	1	9	7	6	4	6	1	9	7	7	6	6	1	9	7	6	4	3	1	9
4	7	6	9	7	1	6	6	3	9	7	9	4	6	1	9	7	6	4	6	1	9
4	3	6	9	7	6	4	7	1	9	7	9	4	6	1	9	7	6	4	6	7	9
4	3	1	9	7	1	6	3	4	9	7	3	4	3	7	9	9	6	4	3	7	9
4	3	6	9	7	6	4	3	1	7	7	4	4	6	1	9	7	6	4	3	1	9
4	9	7	7	7	1	4	9	1	7	7	1	4	7	9	7	7	1	4	7	7	7
4	9	7	9	7	1	4	9	3	9	7	6	4	9	1	9	7	6	4	9	9	9
4	3	7	9	7	6	4	3	1	7	7	7	4	9	1	9	7	6	4	9	1	9

oa1mha	oa1mfe	oa1msd	oa1mdi	oa1msu	oa2mar	oa2mha	oa2mfe	oa2msd	oa2mdi	oa2msu	hpm_O	hpm_Y	hpm_C	MMSE	Cont-O	Cont_Y	Cont-CH
7	6	1	3	1	9	7	6	1	1	1	0.1	168	0.33				
7	6	4	1	6	9	7	4	4	6	6	4	40	0.33				
7	6	4	1	4	9	7	3	4	4	6	4	180	0.33				
7	6	3	3	3	9	7	6	3	3	7	0.33	180	20				
7	6	3	4	4	1	7	6	3	3	7	0.33	172	45				
7	6	4	4	3	9	7	3	4	3	9	0.25	160	0				
7	7	4	4	7	3	7	7	4	3	9	0.1	180	0.3				
7	6	4	4	1	3	7	7	4	3	1	0.25	168	0				
7	6	6	4	7	9	7	6	6	1	4	4	150	18				
7	7	4	4	7	4	7	6	4	1	1	0.33	168	18				
7	6	4	3	7	9	7	6	4	4	3	0.33	186	0				
7	9	4	4	6	9	7	6	4	4	1	0.33	180	4.5				
7	6	4	3	6	9	7	7	4	4	6	0.25	160	0.25				
7	6	4	3	6	9	7	9	4	4	6	0.1	160	0.1				
7	6	6	4	6	6	7	6	6	4	6	8	168	0.25				
7	6	4	4	6	9	7	9	4	4	7	0.1	120	0.1				
7	6	4	3	1	9	7	9	4	4	7	0.25	100	4.5				
7	6	4	3	1	9	7	6	4	4	7	0.33	180	0				
7	6	4	3	1	9	7	1	4	4	7	0.1	168	0.33				
7	6	4	3	9	9	7	6	4	4	1	0.25	168	0.33				
9	6	4	3	1	7	1	1	4	4	7	0.25	100	0.25				
7	6	4	6	4	9	7	1	4	6	7	0.1	180	0.1				
7	9	4	6	1	9	7	1	4	6	7	0.25	80	1				
7	6	6	3	1	9	7	6	6	3	1	0.1	120	0.1				
7	3	4	3	3	9	7	1	4	6	1	24	80	0				
7	6	4	6	3	9	7	6	4	6	7	20	160	27				
7	6	1	6	4	9	7	1	1	6	1	0.1	160	0				
7	6	3	6	3	9	7	6	3	6	7	12	120	31.5				
7	6	3	3	9	9	9	4	3	6	1	0	168	0				
7	6	4	3	6	9	7	6	4	3	7	12	120	18				
1	6	4	6	1	9	7	6	4	6	1	0	100	0				

7	6	6	6	6	9	7	3	6	6	1	20	180	0.1	
7	6	4	6	6	9	7	3	4	3	6	20	168	0	
7	6	4	3	6	9	7	6	4	3	6	4	168	9	
7	6	6	3	6	9	7	6	6	3	1	0.1	180	0	
7	6	6	3	7	9	7	7	6	3	1	0.25	172	0.33	
7	6	4	3	7	9	7	7	4	3	6	0.33	160	0.33	
7	6	4	6	1	9	7	7	4	3	6	16	120	0.25	
7	6	4	6	7	9	7	9	4	3	1	1	168	0.1	
7	6	4	6	1	9	7	9	4	3	1	0.33	100	1	
7	6	4	7	6	9	7	9	4	6	6	0.25	120	0	
7	1	4	7	1	9	7	1	4	6	6	0.1	80	9	
7	1	4	3	1	9	7	1	4	6	6	20	160	9	
7	1	7	7	6	9	7	6	7	6	6	1	160	0.33	
7	6	1	3	6	9	7	6	1	3	1	0.25	168	0.33	
7	6	4	3	7	9	7	6	4	6	4	0.1	120	0.25	
7	6	4	3	1	9	7	6	4	3	1	0	160	0	
7	6	4	7	7	9	9	1	4	7	4	0.1	168	27	
7	6	4	3	1	7	7	6	4	7	1	0.25	120	0	
7	6	3	9	1	9	7	1	3	7	4	0.1	40	40.5	
7	6	3	3	7	9	7	6	3	7	1	0.25	180	0	
7	6	6	3	4	7	7	6	6	9	1	0.33	180	67.5	
7	6	6	3	1	9	7	1	6	3	3	0.25	168	0	
7	6	6	9	3	9	7	1	6	3	3	0.33	180	0.33	
7	6	4	9	9	9	7	1	4	9	3	0.25	172	0.33	
7	6	7	3	1	9	7	6	7	9	1	0.33	160	0.25	
7	6	1	3	1	9	7	6	1	6	1	160	2	0	30
7	6	4	7	7	1	7	6	4	3	6	8	4	0	30
7	6	4	6	1	9	7	6	4	3	6	2	0.33	4.5	30
7	6	3	4	1	1	7	9	3	3	1	180	0.66	0.1	28
7	6	3	3	7	9	7	9	3	4	6	168	0.33	4.5	30
7	6	4	3	6	9	7	3	4	1	1	10	24	0.1	30
7	6	4	1	9	3	7	3	4	1	7	8	24	0	28
7	6	4	3	1	3	7	7	4	4	1	160	8	4.5	30

7	6	3	1	3	9	7	6	3	3	7	3	2	0.25	30			
9	7	3	4	7	9	7	7	3	3	7	180	16	0.33	30			
7	6	6	4	7	9	7	7	6	4	1	24	4	0.25	29			
9	3	6	3	7	9	7	1	6	4	7	12	8	12	30			
1	6	6	3	6	9	7	6	6	3	1	12	32	4.5	30			
7	6	6	3	6	3	7	1	6	4	7	5	1	0	29			
7	6	4	4	6	4	7	1	4	3	1	158	4	45	30			
7	6	4	4	6	9	7	1	4	3	7	10	2	9	29			
7	7	4	4	7	6	7	1	4	3	1	5	1	0	30			
7	6	4	4	7	6	7	6	4	3	1	12	24	54	30			
7	6	9	3	6	7	7	4	9	4	7	160	20	1	29			
7	6	4	4	6	9	7	3	4	4	6	180	32	0.33	28			
7	6	1	4	7	9	7	3	1	4	6	10	12	0.25	30			
7	9	4	6	1	9	7	9	4	4	6	12	20	9	30			
7	6	3	6	1	7	7	9	3	6	6	8	12	4.5	30			
7	6	3	6	7	9	7	7	3	6	6	2	12	9	28			
1	9	3	3	6	9	7	7	3	6	6	10	20	4.5	28			
7	6	4	6	1	9	7	7	4	6	1	156	24	0.1	30			
7	6	3	3	6	7	7	6	3	6	4	168	8	0	30			
7	1	4	6	4	7	7	6	4	6	1	12	16	0.3	29			
1	1	6	6	3	9	7	1	6	3	4	10	24	9	28			
7	6	6	7	4	9	7	6	6	7	1	8	12	4.5	30			
7	6	6	7	3	9	7	1	6	7	3	12	4	0	30			
7	1	4	9	1	9	7	1	4	9	3	180	8	13.5	30			
7	6	6	9	1	7	7	1	6	9	9	160	12	0	28			
7	6	9	9	9	9	7	1	9	9	9	10	16	27	30			
7	3	1	1	3	7	7	4	1	1	7					4	5	3
7	6	1	3	1	1	7	3	1	1	1					3	5	3
7	7	4	1	1	9	7	6	4	3	1					4	8	4
7	6	6	3	7	9	7	7	6	1	7					2	8	3
7	6	4	4	6	9	7	9	4	4	6					8	8	2
7	6	4	3	7	9	7	6	4	3	6					4	7	3
7	6	4	3	7	9	7	9	4	4	4					6	8	4

7	6	4	4	7	9	7	1	4	3	3	4	8	1
7	6	4	3	1	9	7	1	4	4	1	3	6	3
1	6	4	4	7	9	7	4	4	3	7	2	8	3
7	6	4	3	7	9	7	3	4	4	7	8	7	2
7	6	4	3	7	9	7	9	4	3	7	4	8	3
7	9	4	4	7	9	7	6	4	3	1	6	7	4
7	6	4	3	1	3	7	6	4	3	1	4	8	1
7	6	4	3	1	9	7	6	4	3	1	7	5	1
7	6	4	4	6	9	7	6	4	3	7	2	8	2
1	6	4	4	6	9	7	6	4	4	1	7	8	3
7	6	4	3	1	9	7	6	4	4	1	5	8	5
7	6	4	6	1	9	7	9	4	4	1	6	6	2
7	6	6	3	1	9	7	7	6	4	1	4	8	3
7	6	1	3	6	9	7	6	1	4	7	6	8	3
7	6	3	6	6	9	7	6	3	4	6	3	8	2
7	1	6	3	6	9	1	6	6	6	6	4	5	3
7	6	6	6	6	9	7	7	6	3	6	4	7	8
7	6	4	3	1	9	7	6	4	3	6	7	8	4
7	6	9	3	1	9	7	6	9	6	1	7	5	5
9	6	4	6	6	9	7	6	4	6	1	7	8	3
7	6	1	6	3	9	7	7	1	6	6	5	8	3
7	6	4	3	1	9	7	1	4	6	6	7	6	4
7	6	4	7	1	9	9	6	4	6	4	4	8	4
7	6	4	3	9	9	7	6	4	3	1	5	7	5
7	6	3	3	4	9	7	1	3	3	3	4	8	6
7	6	6	3	3	9	7	6	6	7	4	7	7	7
7	1	4	9	4	7	7	1	4	9	9	8	8	5
7	6	6	9	1	7	7	6	6	3	1	5	6	2
7	6	9	3	3	9	7	1	9	9	3	7	8	4
