# Supplementary material for: Age-congruency and contact effects in body expression recognition from point-light displays (PLD)
Source: PeerJ. 2016 Dec 13;4:e2796. doi: 10.7717/peerj.2796 (PMC5157186; doi:10.7717/peerj.2796)
Supplement: Supplemental Information 2 [file peerj-04-2796-s002.pdf]

DATA OF CHILDREN

Exp	ppnr	gender	age	ya1fan	ya1fha	ya1ffe	ya1fsd	ya1fdi	ya1fsu	ch1fan	ch1fha	ch1ffe	ch1fsd
1	1	2	10	9	7	6	4	4	6	9	7	6	4
1	2	1	8	9	7	3	4	3	1	9	7	6	4
1	3	1	9	1	1	6	4	3	7	9	4	6	4
1	4	2	8	9	7	3	3	4	7	9	7	7	4
1	5	2	7	9	7	6	4	3	7	6	7	6	4
1	6	2	7	9	7	3	4	7	4	9	1	6	4
1	7	1	8	9	7	4	6	7	4	9	7	1	4
1	8	1	8	1	7	6	4	3	1	9	7	6	4
1	9	1	8	9	7	6	4	3	1	9	7	6	4
1	10	2	6	9	7	6	4	1	3	9	1	1	4
1	11	1	7	9	7	1	4	1	3	9	1	6	4
1	12	1	7	9	7	6	4	3	1	9	1	4	4
1	13	1	7	1	7	6	4	9	9	4	1	3	4
1	14	2	9	9	7	6	4	9	9	9	7	6	4
1	15	1	9	9	7	1	4	9	1	9	7	6	4
1	16	1	9	6	7	6	4	3	6	9	7	6	4
1	17	1	7	3	7	6	1	6	6	9	7	7	4
1	18	2	6	9	7	1	4	3	6	9	1	1	4
1	19	2	10	9	7	6	4	3	6	9	7	6	4
1	20	1	8	9	1	6	4	6	6	9	1	1	4
1	21	2	9	9	7	6	4	3	6	9	7	6	4
1	22	1	9	9	7	6	4	6	1	9	7	6	4
1	23	1	7	9	7	1	4	6	7	9	1	6	4
1	24	2	7	9	7	9	9	6	7	9	7	7	4
1	25	2	6	9	1	9	4	6	7	9	1	3	4
1	26	1	6	9	7	6	3	3	1	9	9	6	4
1	27	2	7	9	7	6	4	3	1	9	1	6	4
1	28	1	8	9	7	6	3	3	7	9	7	4	4
1	29	2	7	7	1	6	4	4	7	9	7	9	4
1	30	1	8	9	7	9	4	4	7	9	1	4	4
1	31	2	6	9	7	9	4	4	1	9	7	7	4
1	32	1	10	9	1	6	4	3	1	9	7	6	4
1	33	2	6	9	3	6	4	3	1	9	7	6	4
1	34	1	6	3	6	9	6	4	7	9	1	3	4
1	35	2	10	9	7	1	4	3	4	6	1	6	4
1	36	2	7	9	7	1	4	3	4	9	7	1	4
1	37	1	7	9	7	1	4	4	3	7	7	9	4
1	38	1	8	7	7	6	4	4	3	9	1	6	4
1	39	1	9	9	7	6	4	3	9	9	1	1	4
1	40	2	7	9	7	1	4	3	9	9	7	9	4
1	41	1	7	9	7	6	4	4	6	1	7	6	4
1	42	2	8	9	7	6	4	7	6	3	7	6	4
1	43	1	7	9	7	6	4	3	1	9	7	6	4
1	44	1	8	3	7	7	4	7	6	9	6	1	3
1	45	2	6	7	7	6	4	3	1	9	7	6	4
1	46	2	9	9	7	7	4	3	7	9	7	6	4
1	47	1	7	9	7	4	4	3	7	9	7	6	4
1	48	1	8	9	7	3	6	1	1	7	1	6	1

1	49	1	10	9	7	6	4	1	7	1	7	6	4
1	50	1	7	9	7	1	4	3	7	9	1	6	4
1	51	2	7	9	7	1	4	3	7	9	1	9	4
1	52	2	7	9	7	1	4	1	7	3	7	1	4
1	53	1	9	3	7	1	7	9	7	7	7	9	6
1	54	2	7	9	4	1	4	9	4	9	7	9	4
1	55	2	7	9	7	9	4	9	4	9	7	9	4
1	56	1	7	7	7	9	1	6	3	1	1	9	3
1	57	2	6	9	1	9	4	6	3	9	7	9	4
1	58	1	7	9	7	9	9	6	9	9	7	6	4
1	59	1	7	9	7	7	4	6	9	3	7	1	4
1	60	1	7	9	7	6	4	3	1	9	7	6	4
1	61	1	7	3	7	9	3	6	6	7	4	1	4
1	62	2	7	9	7	6	4	3	1	9	7	1	4
1	63	1	8	7	7	6	3	3	6	1	7	1	4
1	64	2	7	9	7	6	4	6	6	9	7	6	4
1	65	2	6	9	7	3	6	4	6	9	7	1	4
1	66	2	7	9	7	6	4	4	6	9	1	1	4
1	67	2	10	3	7	6	4	3	7	9	1	1	4
1	68	1	9	7	1	1	4	4	1	9	7	6	4
1	69	2	8	9	1	6	4	3	7	9	7	6	4
1	70	1	7	9	7	6	6	3	7	3	7	6	4
1	71	1	6	9	7	9	4	4	7	9	1	1	6
1	72	1	9	9	7	7	4	3	7	7	7	1	4
1	73	2	10	9	7	6	4	4	1	9	1	6	4
1	74	2	8	9	7	6	4	4	7	9	7	6	4
2	1	2	7	9	7	1	4	7	3	9	7	4	4
2	2	1	10	9	7	6	4	1	9	9	7	6	4
2	3	2	9	9	7	6	4	1	7	9	1	3	4
2	4	1	7	9	7	1	4	9	3	4	1	3	4
2	5	1	7	9	7	7	4	9	3	4	7	6	4
2	6	2	7	9	7	6	4	6	3	9	1	6	4
2	7	2	7	9	1	6	4	3	6	9	1	6	4
2	8	2	9	9	9	9	4	6	6	9	7	6	4
2	9	2	7	9	7	6	9	6	6	1	7	1	4
2	10	1	10	9	7	1	4	6	6	1	1	6	4
2	11	1	6	9	7	6	4	6	6	6	1	1	0
2	12	2	10	9	7	6	4	3	1	9	7	6	4
2	13	1	9	9	7	6	4	3	9	9	7	6	4
2	14	2	10	9	7	6	4	3	1	9	7	6	4
2	15	2	7	9	7	6	4	6	9	9	7	1	4
2	16	1	8	9	7	6	4	4	1	9	7	6	4
2	17	2	6	9	7	6	4	3	4	3	9	9	4
2	18	2	6	4	7	1	4	3	4	9	7	6	4
2	19	1	10	9	7	6	4	3	1	9	7	6	4
2	20	2	10	9	9	1	4	3	4	9	7	6	4
2	21	1	8	9	7	1	4	3	1	9	6	9	4
2	22	1	7	9	6	1	4	3	4	9	7	6	4
2	23	1	7	9	6	1	4	4	4	9	3	9	4
2	24	2	6	1	3	1	4	4	3	7	1	7	3

2	25	2	8	9	7	7	4	4	1	3	1	7	4
2	26	2	7	9	7	6	4	3	3	9	7	7	4
2	27	1	9	9	7	1	4	4	3	9	1	1	4
2	28	1	6	6	7	6	6	4	1	9	7	6	6
2	29	2	7	9	7	1	4	3	1	9	7	1	9
2	30	2	7	9	7	1	4	4	1	9	7	1	7
2	31	2	6	9	1	1	4	3	3	7	7	6	4
2	32	1	9	9	7	6	4	3	7	9	1	6	4
2	33	1	6	9	1	1	4	4	7	3	1	1	4
2	34	1	7	9	7	1	4	3	7	9	1	1	1
2	35	1	7	9	7	6	4	3	7	9	7	6	4
2	36	1	8	9	7	1	4	4	7	9	1	1	4
2	37	1	8	9	7	3	4	7	1	9	7	6	4
2	38	2	6	9	7	6	4	3	6	9	7	6	4
2	39	2	6	9	7	1	4	7	6	9	7	1	4
2	40	2	8	9	7	6	4	3	6	9	7	6	4
2	41	2	6	9	7	7	4	3	6	9	7	1	4
2	42	1	7	9	7	6	4	3	1	9	9	6	4
2	43	1	7	9	1	6	4	3	6	9	7	6	4
2	44	2	7	6	7	6	4	3	6	9	7	6	4
2	45	1	6	9	7	6	4	1	1	7	6	6	4
2	46	2	6	9	1	3	4	1	6	3	1	1	4
2	47	2	8	9	7	1	4	1	9	9	7	1	4
2	48	1	9	9	7	6	4	3	9	9	7	6	4
2	49	1	9	9	7	6	4	3	4	9	1	6	4
2	50	2	7	7	7	1	4	9	4	7	7	6	4
2	51	1	6	3	7	1	4	9	3	3	7	6	3
2	52	2	6	9	7	1	4	9	3	9	7	7	4
2	53	2	7	9	7	1	4	3	7	9	1	7	4
2	54	2	8	9	7	1	4	3	7	9	7	6	4
2	55	2	6	7	7	9	6	6	7	7	1	9	4
2	56	1	9	9	7	7	4	3	7	9	7	6	4
2	57	2	8	9	7	6	4	6	7	9	7	9	4
2	58	1	6	9	7	4	1	3	7	6	1	9	4
2	59	2	6	3	7	3	4	6	7	3	7	9	3
2	60	2	7	7	7	1	3	6	1	9	1	4	4
2	61	1	6	9	1	6	4	4	6	9	7	6	4
2	62	1	10	3	7	6	4	4	6	9	7	6	4
2	63	2	7	9	7	1	4	3	6	9	7	3	6
2	64	2	8	9	7	1	4	3	1	9	7	7	4
2	65	2	7	9	7	1	4	3	6	9	7	6	4
2	66	1	6	9	7	7	4	4	6	9	1	7	4
2	67	1	9	9	7	9	4	3	6	9	7	9	4
2	68	2	7	9	7	6	4	3	6	9	1	9	4
2	69	1	9	9	7	6	4	4	1	7	7	6	4
2	70	2	6	9	7	3	4	4	9	9	7	6	4
2	71	2	6	9	7	6	4	4	9	9	9	9	6
2	72	1	6	9	7	4	4	7	9	9	7	9	4
2	73	2	9	9	7	6	4	3	4	9	6	6	4
2	74	2	8	9	7	6	4	7	1	9	7	6	4

2	75	1	6	9	7	6	4	3	1	6	7	6	4
2	76	1	8	9	3	1	3	1	1	9	7	6	4
2	77	1	6	9	7	1	4	1	4	3	7	6	4
2	78	1	8	9	7	6	4	9	4	9	7	6	4
2	79	2	9	9	7	6	4	3	1	9	7	6	4
2	80	1	7	9	7	1	4	9	1	9	7	1	4
2	81	2	8	7	7	6	4	9	3	9	7	6	4
2	82	1	7	9	7	6	4	3	3	9	7	6	4
2	83	1	8	9	7	1	4	6	3	9	1	1	4
2	84	2	7	9	9	6	4	3	7	7	1	6	4
2	85	2	6	9	7	7	4	6	1	9	7	1	4
2	86	2	9	9	7	6	4	3	7	9	7	6	4
2	87	2	6	3	7	9	4	6	7	6	7	1	4
2	88	1	9	9	7	4	6	6	7	9	7	6	4
2	89	2	8	9	1	6	4	4	7	9	1	1	4
2	90	2	7	9	7	3	4	4	1	9	1	6	4
2	91	1	7	9	7	1	4	3	6	9	1	6	4
2	92	1	10	9	7	1	4	3	6	9	1	6	4
2	93	2	6	9	7	6	4	3	1	3	1	1	4
2	94	1	8	7	7	1	6	4	6	9	1	6	4
2	95	2	6	9	7	7	4	3	6	9	7	1	4
2	96	2	6	9	7	6	4	4	6	7	7	1	4
2	97	2	8	9	7	9	4	3	1	9	7	6	4

ch1fdi	ch1fsu	oa1fan	oa1fha	oa1ffe	oa1fsd	oa1fdi	oa1fsu	hpm_OA	hpm_YA	hpm_CH	Cont_OA	Cont_YA	Cont_CH
3	1	9	7	6	4	6	9	28	0	100			
6	1	9	7	6	4	1	1	28	12	120			
3	4	3	7	6	4	1	1	0	0	100			
7	4	9	7	9	4	7	4	0	20	100			
3	4	6	7	1	4	7	4	0	20	100			
1	3	1	7	6	4	9	1	0	4	210			
9	3	9	7	1	6	9	3	0	0.1	220			
7	1	9	7	4	4	3	1	16	0.25	206			
3	1	9	7	6	4	6	1	26	0.33	100			
6	1	4	7	6	4	6	1	24	16	100			
6	3	9	7	6	4	6	3	14	1	100			
6	1	9	1	6	4	3	1	0	0.33	210			
6	1	4	7	4	4	6	6	0	0.25	155			
3	1	1	6	4	6	6	6	0	0.1	80			
3	6	9	7	7	4	4	6	28	20	100			
3	6	9	7	6	4	4	6	1	1	80			
6	6	9	7	1	4	4	7	1	0.25	210			
6	6	9	1	3	4	3	7	3	0.1	160			
3	1	3	7	6	4	4	7	16	0	200			
3	1	9	7	6	4	4	1	4	0.1	80			
4	1	9	7	9	4	4	1	4	0.25	100			
4	1	9	7	7	4	7	7	0	0.1	120			
4	7	9	7	1	4	7	7	1	0.25	210			
4	7	9	7	3	4	7	7	16	0.33	100			
4	1	7	3	9	4	1	1	1	0.25	100			
3	7	9	7	3	4	1	7	3	0.1	80			
4	7	6	7	3	4	1	9	0	0.25	200			
4	9	9	7	3	4	9	1	1	0.1	88			
4	9	9	7	1	4	9	9	2	24	92			
7	9	9	7	1	4	3	9	1	20	180			
7	4	9	7	6	4	9	4	28	0.1	132			
1	1	9	7	6	4	9	1	12	12	156			
1	1	9	7	6	4	6	4	16	0	200			
3	4	3	1	1	4	6	3	1	12	140			
3	4	7	1	6	4	6	3	0	0	132			
3	3	3	4	7	4	6	6	0.5	20	210			
9	3	9	7	7	4	6	6	1	20	88			
9	3	7	7	7	4	6	6	0	4	100			
3	6	3	7	6	4	6	6	1.5	0.1	200			
3	6	7	7	6	4	4	6	2	4	210			
6	6	9	7	9	4	4	6	2	0.33	188			
6	6	9	7	9	4	4	7	3	0.33	150			
6	6	9	7	6	4	4	7	0	0.25	98			
6	6	9	7	9	1	4	7	28	0.1	92			
6	1	9	7	3	4	3	1	0	0.25	80			
6	6	1	7	6	4	3	1	4	4	180			
4	7	3	1	3	4	4	7	28	0.33	180			
4	1	9	7	6	4	4	7	26	0.33	156			

3	7	9	7	3	4	7	7	2	0.33	140			
4	7	9	1	7	4	7	1	1	0.25	80			
4	7	9	7	7	7	7	1	28	0.1	132			
4	7	6	7	6	4	1	9	20	8	92			
7	7	7	7	7	4	1	9	0	0.1	92			
3	9	1	1	6	9	1	4	0	0.25	98			
7	9	9	7	6	4	9	4	10	20	156			
1	4	9	7	6	6	9	1	24	0.1	200			
1	4	3	7	6	3	9	1	2	12	200			
9	3	6	7	6	4	9	3	18	0	200			
9	3	1	7	7	4	3	3	18	1	92			
9	3	7	7	1	4	6	3	0	0.25	80			
6	6	3	7	6	3	3	6	16	0.1	92			
3	6	9	7	1	4	3	6	18	0	104			
6	6	6	7	6	4	6	6	18	0.1	206			
6	6	9	7	1	4	6	6	24	0.25	156			
6	6	9	7	1	4	6	1	16	0.1	92			
6	6	9	1	9	6	6	7	0	20	80			
4	7	9	7	9	4	3	7	16	20	100			
4	7	1	7	9	4	4	1	12	4	92			
4	7	9	7	6	4	3	7	14	0.1	92			
4	1	9	7	9	4	4	1	16	0.25	122			
4	7	3	7	6	4	3	7	16	0.33	156			
4	1	9	7	6	4	4	1	18	16	210			
4	7	7	7	6	4	4	7	1	1	202			
4	7	9	7	6	4	4	1	28	2	200			
4	6	6	7	9	4	1	1				4	8	8
4	1	9	7	6	4	3	1				8	8	8
7	1	4	7	6	4	3	1				7	5	8
7	1	9	7	9	4	1	7				4	6	8
7	6	9	7	6	4	3	1				8	8	8
3	6	9	7	6	4	7	7				4	7	8
1	1	4	7	6	4	3	1				1	7	8
3	6	9	7	6	4	3	7				4	7	8
1	7	9	7	1	4	7	6				4	6	8
3	7	9	7	6	4	3	1				8	4	8
1	7	1	6	1	4	1	6				2	8	8
3	1	9	7	6	4	3	1				1	8	7
3	1	1	7	4	4	1	1				4	7	8
3	1	9	7	6	4	3	6				7	4	7
3	1	6	7	6	4	6	1				7	4	8
9	1	6	7	6	4	6	6				4	7	8
9	7	6	7	3	4	6	4				3	7	8
3	7	6	7	3	4	6	1				6	3	8
3	1	9	7	6	4	3	1				8	7	8
3	1	9	7	6	6	6	4				7	7	8
6	7	9	7	6	4	6	9				7	7	8
4	1	9	7	6	4	9	9				7	7	8
6	1	9	7	6	4	9	1				5	7	8
6	9	3	1	6	4	3	1				8	8	7

6	1	9	7	3	4	9	3	4	7	8
4	9	9	7	6	4	7	3	3	5	8
3	1	9	9	6	4	7	1	7	7	8
4	1	3	7	6	4	7	6	3	5	8
4	4	3	1	9	4	7	6	1	8	8
4	4	9	7	9	4	3	6	2	8	8
3	1	6	1	1	4	4	1	2	8	8
3	4	9	7	6	4	4	1	7	6	7
4	1	6	7	7	4	4	1	2	1	8
7	3	9	3	4	6	3	1	3	7	8
7	3	9	7	1	4	4	1	7	7	8
7	1	6	1	6	4	4	6	7	8	8
1	3	7	7	3	4	4	6	2	3	8
1	3	9	7	7	4	4	6	3	6	8
1	6	7	7	1	4	4	1	6	3	8
9	1	9	7	6	4	1	7	3	4	8
6	6	9	7	1	4	1	7	4	7	8
6	6	9	7	6	4	3	1	8	7	8
9	6	9	7	1	4	1	7	4	4	8
3	6	7	1	6	4	1	7	1	7	8
4	1	9	7	9	4	1	1	8	5	8
4	6	9	1	9	4	1	1	3	4	8
4	7	7	7	1	4	7	7	2	6	8
3	1	9	7	1	4	7	1	7	8	8
3	7	9	7	1	4	7	7	4	6	8
4	7	9	6	1	3	7	4	8	6	8
4	1	6	7	6	4	3	4	1	8	8
4	1	3	7	1	4	7	9	2	6	7
4	7	9	7	1	1	9	1	4	8	8
7	7	9	7	6	4	3	9	1	7	8
7	7	3	1	1	6	9	1	1	6	8
3	7	9	7	7	4	9	1	3	7	8
3	9	9	7	6	4	9	1	8	6	7
1	9	9	7	7	4	6	3	3	6	8
1	4	6	7	6	4	6	1	8	6	8
9	4	9	1	9	7	6	1	2	7	8
9	4	7	9	6	4	6	3	2	4	8
3	3	9	7	9	4	6	7	3	3	8
9	3	7	7	6	4	4	7	4	7	8
6	1	9	7	6	4	4	7	7	7	8
6	3	9	7	3	4	4	7	4	8	8
6	6	3	7	9	9	4	7	1	7	8
6	6	9	7	6	4	3	1	1	7	8
6	6	9	7	6	4	3	6	6	6	8
6	6	9	7	6	4	3	6	8	5	8
6	6	6	7	1	4	4	6	8	5	8
4	6	9	7	1	4	4	6	3	5	7
4	6	9	7	1	4	4	6	4	8	8
3	7	9	7	6	4	3	9	4	7	8
4	7	9	7	7	4	7	1	7	6	8

4	7	3	1	6	4	7	1	8	8	8
4	1	9	1	6	4	3	1	8	7	8
4	7	9	7	7	4	1	4	5	7	8
7	7	6	7	6	4	1	3	4	8	8
3	7	9	7	7	4	9	1	2	6	8
3	9	7	1	9	4	9	1	7	7	8
1	9	3	7	9	4	6	1	4	7	8
9	9	9	7	6	4	6	7	7	5	7
6	4	9	1	6	4	6	1	7	7	8
3	4	9	7	6	4	6	7	7	6	8
3	3	9	7	9	4	6	1	8	5	8
3	3	9	7	9	4	6	1	4	5	8
6	6	7	7	1	4	6	7	2	6	8
3	6	9	7	6	4	3	1	7	6	8
6	6	9	7	6	4	4	7	6	5	8
6	1	9	7	6	4	4	6	8	8	8
4	6	6	7	6	4	4	1	8	8	8
3	7	9	7	1	4	3	6	3	5	8
4	1	9	7	1	4	4	1	3	7	8
4	7	3	7	1	4	4	6	8	5	8
4	7	6	7	1	4	4	1	5	3	8
3	7	7	7	1	4	3	6	5	7	8
3	1	9	7	6	4	3	1	7	4	7
